# Cytokine profiles as predictors of HIV incidence using machine learning survival models and statistical interpretable techniques

**DOI:** 10.1038/s41598-024-81510-y

**Published:** 2024-12-02

**Authors:** Sarah Ogutu, Mohanad Mohammed, Henry Mwambi

**Affiliations:** 1 School of Mathematics, Statistics and Computer Science, University of KwaZulu-Natal, Pietermaritzburg, 3201 South Africa; 2 School of Nursing and Public Health, University of KwaZulu-Natal, Pietermaritzburg, 3201 South Africa

**Keywords:** Cytokine profiles, HIV incidence, Random survival forest, Survival support vector machine, SHAP values, C-index, Integrated Brier score, Predictive markers, HIV infections

## Abstract

HIV remains a critical global health issue, with an estimated 39.9 million people living with the virus worldwide by the end of 2023 (according to WHO). Although the epidemic’s impact varies significantly across regions, Africa remains the most affected. In the past decade, considerable efforts have focused on developing preventive measures, such as vaccines and pre-exposure prophylaxis, to combat sexually transmitted HIV. Recently, cytokine profiles have gained attention as potential predictors of HIV incidence due to their involvement in immune regulation and inflammation, presenting new opportunities to enhance preventative strategies. However, the high-dimensional, time-varying nature of cytokine data collected in clinical research, presents challenges for traditional statistical methods like the Cox proportional hazards (PH) model to effectively analyze survival data related to HIV. Machine learning (ML) survival models offer a robust alternative, especially for addressing the limitations of the PH model’s assumptions. In this study, we applied survival support vector machine (SSVM) and random survival forest (RSF) models using changes or means in cytokine levels as predictors to assess their association with HIV incidence, evaluate variable importance, measure predictive accuracy using the concordance index (C-index) and integrated Brier score (IBS) and interpret the model’s predictions using Shapley additive explanations (SHAP) values. Our results indicated that RSFs models outperformed SSVMs models, with the difference covariate model performing better than the mean covariate model. The highest C-index for SSVM was 0.7180 under the difference covariate model, while for RSF, it reached 0.8801 under the difference covariate model using the log-rank split rule. Key cytokines identified as positive predictors of HIV incidence included TNF-A, BASIC-FGF, IL-5, MCP-3, and EOTAXIN, while 29 cytokines were negative predictors. Baseline factors such as condom use frequency, treatment status, number of partners, and sexual activity also emerged as significant predictors. This study underscored the potential of cytokine profiles for predicting HIV incidence and highlighted the advantages of RSFs models in analyzing high-dimensional, time-varying data over SSVMs. It further through ablation studies emphasized the importance of selecting key features within mean and difference based covariate models to achieve an optimal balance between model complexity and predictive accuracy.

## Introduction

Cytokines are signaling molecules generated by immune cells reacting to infections, inflammation, or stimuli^1^. They play crucial roles in modulating immune responses and maintaining homeostasis in the body. Recent studies have suggested that cytokine profiles may be valuable biomarkers for predicting HIV incidence, as they reflect the dynamic interplay between host immune responses and viral infection^2^. Traditional statistical methods, such as the Cox proportional hazards (PH) model, Log-normal and Weibull have been extensively employed to analyze survival data in HIV research^3^. However, these methods may have limitations in handling high-dimensional data, capturing non-linear relationships between predictors, and accommodating violations of the proportional hazards assumption^4^. Machine learning (ML) survival data models emerge as potentially alternatives owing to their flexibility and absence of overly restrictive priori assumptions^5^. They can adeptly handle intricate interactions, a challenge for conventional statistical methods, and further can flexibly incorporate all available information^6^. Moreover, ML approaches can be especially effective in scenarios with few observations and many predictors^5^. Random survival forest (RSF) and survival support vector machine (SSVM) models offer a promising alternative for analyzing survival data^7–14^, particularly in the context of high-dimensional longitudinal data such as multiple cytokine profiles as in the present study. By leveraging ensemble learning techniques and decision trees, RSF models can capture complex interactions between predictors and provide robust predictions of survival outcomes^4^. In contrast, Survival Support Vector Machines (SSVM) use kernel functions to account for complex, non-linear relationships between features and survival outcomes^15^.

In the primary analysis of the CAPRISA 004 dataset conducted by Karim et al.^16^ and Mansoor et al.^17^ the traditional Cox PH model was employed. However, they adjusted for potentially significant baseline covariates and did not use any cytokine profile due to the high dimensionality and complexity of the dataset. In other studies that utilized the same dataset with different statistical models, a few cytokine profiles were selected for their analysis^18^ (12 selected cytokines),^19^ (13 selected cytokines),^20^ (10 selected cytokines). The selection strategy was to a certain extent disadvantageous as other potential significant variables were excluded from the analysis. Our study therefore, proposes the use of RSF and SSVM model as an alternative approach for analyzing HIV survival data, particularly in the context of high-dimensional time-varying cytokine profiles.

Our objective is to employ SSVM and RSF models to identify cytokine profiles as potential predictors of HIV incidence. We accomplished that by constructing models that incorporate derived cytokine variables from longitudinal measurements along with baseline variables as covariates in the models by using the average and the difference of the first and last measurements within an individual’s profile for all cytokines. We implemented RSF models using both log-rank and log-rank score split rules and evaluated their performance using measures which include the concordance index (C-index) and integrated Brier scores (IBS). Additionally, we assessed variable importance using permutation-based methods^21,22^ and interpreted the model’s predictions using SHAP values^23–25^. To optimize model complexity and predictive accuracy, we conducted an ablation study by progressively adding top-ranked features based on variable importance (VIMP) scores and analyzing the impact on model performance. Moreover, SSVM was fitted to compare the models’ predictive performances through C-index because it offers a user-friendly experience and demonstrates high efficiency, particularly when handling extensive datasets^26^. The incorporation of cytokine profiles into predictive models enhances our understanding of the complex interactions between host immune responses and HIV infection dynamics.

## Material and methods

### Dataset

The dataset utilized in this study was obtained from the Centre for the AIDS Programme of Research in South Africa (CAPRISA 004)^16^, which conducted a two-arm, double-blinded, randomized trial involving the placebo and tenofovir groups. The trial targeted HIV negative, sexually active women aged 18–40 years in South Africa over a 30 month period, comprising an 18 month accrual phase followed by a 12 month follow-up period. This dataset consisted of longitudinally measured cytokines (48 in total) and 46 baseline characteristics collected from 812 women, among whom 96 acquired HIV infections. The cytokines measurements were obtained from stored plasma samples and cervicovaginal lavage specimens from cases and control groups. High-dimensional datasets such as this often contain noisy or uninformative variables. Therefore, data cleaning was an imperative initial step before modeling. This study implemented a pre-processing procedure on the dataset to eliminate categorical variables with inadequate levels and excessive missing values. The data preparation and subsequent statistical analyses were conducted using *R* (version R-4.4.1)^27^ . Following the pre-processing stage, 25 baseline and 48 cytokine covariates were retained for further analysis. Given the time-dependence nature of cytokine profiles, their information was incorporated in two ways: first, by averaging all measurements throughout the follow-up to capture their average effect, and secondly, by calculating the difference between the last and first measurements to model the effect of change.

### Machine learning approaches

Two distinct models (mean and difference models) were fitted for the SSVM and RSF (employing the log-rank and log-rank-split rules). The mean model incorporated baseline variables alongside cytokine profiles, utilizing the mean value of the cytokine measurement as a covariate. Conversely, the difference model included baseline variables in conjunction with cytokine profiles, with the cytokine covariate being the difference between the last observed cytokine value and the initial measurement. Before implementing the RSF and SSVM analysis, the dataset for each model was partitioned into a training set, comprising 80% of the dataset, and a test set, comprising the remaining 20% of the dataset.

#### Survival support vector machine (SSVM)

The survival support vector machine (SSVM) is an extension of the conventional support vector machine (SVM) tailored for right-censored time-to-event data^28^. It presents a significant advantage due to its ability to accommodate intricate, non-linear associations between features and survival outcomes through the kernel trick^29^. Through this mechanism, a kernel function adeptly transforms input features into higher-dimensional spaces, enabling the depiction of survival via a hyperplane^30^. This versatility renders SSVMs highly adaptable and suitable for diverse datasets^31^.

A kernel function serves as a mechanism for transforming input data into a suitable format for further processing^32^. The function transforms the training data, enabling a nonlinear decision boundary to manifest as a linear equation in a higher-dimensional space. The standard kernel function is given by Eq. (1)

1 $$\begin{aligned} K(\bar{x})= {\left\{ \begin{array}{ll} 1 & \hspace{0.5cm} \text {if}\hspace{0.2cm}||\bar{x}|| \le 1\\ 0 & \hspace{0.5cm} \text {Otherwise} \end{array}\right. } \end{aligned}$$

where $||\bar{x}||$ is the norm of the vector $\bar{x}$. Some popular kernel functions used in SVMs include polynomial, sigmoid, linear, additive kernels, and radial basis function (RBF)^33^. The selection of a Kernel function relies on the data’s characteristics and the intended level of complexity within the model.

Within the SVM domain, survival data analysis unfolds through three distinct approaches. First, the regression approach^34^, which draws from the concept of support vector regression (SVR) idea^35^. Regression-based SSVMs involve directly modeling survival times (or a related quantity like log survival times). This approach seeks to ascertain a function that predicts observed survival times as continuous outcomes ($y_i$) by leveraging covariates ($x_i$). Second, the ranking approach views survival analysis via SVMs as a classification task^36^, where the objective is to predict ordinal risk ranks among individuals^37^. Finally, the hybrid approach merges elements of regression and ranking approaches in the SSVM problem^38^.

The hybrid optimization problem is given by Eq. (2)

2 $$\begin{aligned} \begin{aligned} \min \limits _{\psi ,b,\epsilon ,\zeta ,\zeta ^*} \hspace{0.5cm}&\frac{1}{2}||\psi ||^2+\gamma \sum _{i=1}^{n}\varepsilon _i+\mu \sum _{i=1}^{n}(\zeta _i-\zeta _i^*)\\ \text {Subject to} \hspace{0.5cm}&\left\langle \psi ,F(x_i) \right\rangle -\left\langle \psi ,F(x_{\bar{ j}(i)})\right\rangle \ge y_i-y_{\bar{j}(i)}-\varepsilon _i,\\&y_i-\left\langle \psi ,F(x_i)\right\rangle -b\le \zeta _i,\\&\sigma _i (\left\langle \psi ,F(x_i)\right\rangle +b-y_i) \le \zeta _i^*,\\ \text {and} \hspace{0.5cm}&\varepsilon _i,\zeta _i,\zeta _i^* \ge 0\\ \end{aligned} \end{aligned}$$

where $i=1,...,n$, $\varepsilon _i$, $\zeta _i$ and $\zeta _i^*$ are error constraints, *b* is the bias, $\psi$ and $\sigma _i$ are the weight vectors, $\gamma$ and $\mu$ are regularization parameters and $F(x_i)$ is the feature vector of $x_i$ or $x_j$. The problem is equivalent to maximizing the concordance index as defined by Van Belle et al.^36^ over comparable pairs for a given prediction function *u* (Eq. 3) as

3 $$\begin{aligned} CI_n(u)=\frac{1}{n(n-1)}\sum _{v_{ij}=1}I[(u(x_i)-u(x_j))(t_i-t_j)] \end{aligned}$$

where $I(a)=1$ if $a>0$ and $I(a)=0$ otherwise. *I*(.) is the indicator function for comparable pair $v_{ij} (x_i, x_j)$ and survival times $(t_i, t_j)$. $n(n-1)$ is the total number of possible pairs in the dataset. We implemented the SSVM, particularly the hybrid approach, using the *R* package *survivalsvm*^39^.

#### Random survival forests (RSF)

The random survival forests (RSF) represent an ensemble tree method tailored to analyze right-censored survival data^40^. Derived from Breiman’s random forests (RF) methodology^41^, RSF extends its capabilities to handle high-dimensional data effectively^42,43^. In particular, in scenarios with complex and non-linear relationships between dependent and independent variables, RSF excels even when covariates violate the proportional hazards (PH) assumption^44^. One of the notable strengths of RSF is its independence from specific model assumptions, unlike the Cox PH model. Thus, while the Cox PH is a semi-parametric model, the RSF is inherently non-parametric. It endeavors to adeptly capture the underlying data patterns, particularly in situations with limited survival data. Additionally, RSF exhibits resilience to high-dimensional data and is robust against outliers in the explanatory variables^42^. The RSF methodology employs two randomization steps to grow the tree: bootstrapping to randomly select cases and the random selection of covariate subsets for the tree’s node splitting. These steps aid in reducing the correlation between individual trees in the forest^41^. RSF was implemented using the *randomForestSRC* package in *R*^45^.

We employed the RSF algorithm introduced by Ishwaran et al.^40^ depicted below:

- B bootstrap samples were drawn from the original dataset (training data), ensuring that each bootstrap excluded approximately 37% of the samples, thereby creating out-of-bag (OOB) data.
- A survival tree was developed for every bootstrap sample. At each node within the tree, *p* cytokine/baseline covariates were randomly selected. The node was then split using the candidate variable that optimally maximizes the survival difference between its daughter nodes.
- The tree was expanded to its maximum size while ensuring that each terminal node contained no fewer than $d_0 > 0$ distinct HIV incidences.
- A cumulative hazard function (CHF) was computed for each individual tree. Then, the CHFs across all ntrees were averaged to derive the ensemble CHF.
- By Utilizing the OOB data, the prediction error for the ensemble CHF was determined.

Ultimately, the survival tree reached a point of saturation wherein no further daughter nodes could be created. The terminal nodes in a saturated tree were considered the most extreme nodes; we denoted them by $\Upsilon$. Let $(T_{1,h}, \theta _{1,h}), ..., (T_{n(h),h}, \theta _{n(h),h})$ represent the survival times and the censoring information (0,1) for the *n*(*h*) individuals (cases) in a terminal node $h \in \Upsilon$. An individual *i* is considered right-censored at time $T_{i,h}$ if $\theta _{i,h} = 0$; conversely, if $\theta _{i,h} = 1$, the individual is deemed to have HIV infection at time $T_{i,h}$. Let $t_{1,h}< t_{2,h}< ... < t_{n(h),h}$, $d_{l,h}$ denote the distinct ordered event times and $M_{l,h}$ is the risk set at time $t_{l,h}$ while $d_{l,h}$ is the number of infections at event time $t_{l,h}$. Therefore, the CHF estimate for node *h* was determined using the Nelson-Aalen estimator^46^ (Eq. 4) as follows

4 $$\begin{aligned} \hat{H}_h(t)=\sum _{t_{l,h\le t}}\frac{d_{l,h}}{M_{l,h}} \end{aligned}$$

All cases within node *h* exhibit identical CHFs. The CHF for *i* given a vector of cytokines and baseline variables as a covariate ${C_i}$ was estimated for a single tree as (Eq. 5)

5 $$\begin{aligned} \hat{H}_h (t|{C_i})=\hat{H}_h(t) \quad for\,{i} \in n(h) \end{aligned}$$

To derive an ensemble CHF, the average of the ntrees was computed. The bootstrap ensemble CHF for an observation i was determined by Eq. (6):

6 $$\begin{aligned} \hat{H}_e(t|{C_i})=\frac{1}{B}\sum _{b=1}^{B}\hat{H}_b(t|{C_i}) \end{aligned}$$

Every tree within the forest was developed utilizing an independent bootstrap sample (Eq. 7). Let

7 $$\begin{aligned} I_{i,b}= {\left\{ \begin{array}{ll} 1 & \hspace{2cm} \text {if}\, i\, \text {is an OOB case for}\, b \\ 0 & \hspace{2cm} \text {Otherwise} \end{array}\right. } \end{aligned}$$

Subsequently, the OOB ensemble CHF for i was computed as (Eq. 8)

8 $$\begin{aligned} \hat{H}_e^*(t|{C_i})= \frac{\sum _{b=1}^{B}I_{i,b}\hat{H}_b^*(t|{C_i})}{\sum _{b=1}^{B}I_{i,b}} \end{aligned}$$

thus, $\hat{H}_e^*(t|{C_i})$ represents an average across bootstrap samples where i is from OOB case.

We employed two split rules;

- Log-rank split rule.
  The log-rank split rule serves as a criterion for node separation, aiding in the identification of the optimal split for a given node^47^. Consider a split at node *h*. At this node, the available data was presented as $(C_1, T_1, \theta _1), ..., (C_{n(h)}, T_{n(h)}, \theta _{n(h)})$, where $C_i$, $T_i$ and $\theta _i$ represent the $i^ {th}$ predictor, survival duration, and censoring status respectively. When a split was made using covariate *c* and its splitting value *a* the survival difference between any two daughter nodes was computed through the log-rank statistic^47^, expressed as
  9 $$\begin{aligned} L(c,a)=\frac{\sum _{i=1}^{n(h)}\left( d_{i1}-Y_{i1}\frac{d_i}{Y_i}\right) }{\sqrt{\sum _{i=1}^{n(h)} \frac{d_i}{Y_i}\left( 1-\frac{Y_{i1}}{Y_i}\right) \left( \frac{Y_i-d_i}{Y_i-1}\right) d_i}} \end{aligned}$$
  Eq. (9) quantifies the degree of separation between the two daughter nodes. Where $d_{i1}$ is the number of events from daughter node 1, $Y_{i1}$ is the corresponding number at risk, $d_i$ and $Y_i$ are the total number of events and number at risk between the two daughter nodes.The optimal split was determined by identifying the largest difference between the two daughter nodes^40^, which corresponds to the highest value of the |*L*(*c*, *a*)|. This process was iterated at each node until reaching the terminal node.
- Log-rank score split rule.
  The log-rank score splitting rule evolved from the log-rank split rule^48^. If we consider $(r=r_1,r_2,...,r_n)$ as a ranking vector for survival times $T_l,\theta _l=((T_1,\theta _1),(T_1,\theta _1),...,(T_n,\theta _n))$ and $g=g(T_l,\theta _l)=(g_1(r),g_2(r),...,g_n(r))$ as the ranked score vector then, the ranks for each survival time $T_l$ were determined based on an ordered predictor *C* ensuring that $C_1<C_2<,...,<C_n$^49^. For each time $T_l$ the rank was calculated from Eq. (10) given by
  10 $$\begin{aligned} g_l=\theta _l-\sum _{k=1}^{\lambda _l}\frac{\theta _k}{n-\lambda _k+1} \end{aligned}$$
  where $\lambda _l$ was the number of individuals who were HIV infected or were censored before at time $T_l$ $(t:T_t\le T_k)$. Assume $\bar{g}$ and $s_g^2$ denote the sample mean and variance for $g_l$ respectively, where $l=1,2,...,n$. The formula for the log-rank score test statistic is expressed in Eq. (11) as
  11 $$\begin{aligned} S(c,a)=\frac{\sum _{c_l\le a}g_l-n_i\bar{g}}{\sqrt{n_1[1-\frac{n_1}{n}]}s_g^2} \end{aligned}$$
  This split rule quantifies the degree of separation between nodes by |*S*(*c*, *a*|), where the optimal split was determined by the maximum value between *c* and *a* and $n_1$ is the number of cases in daughter node 1.

### Statistical comparison measures (performance evaluation)

#### Concordance index (C-Index)

To assess prediction error, we utilize Harrell’s concordance index^50^. This index quantifies the probability that the case failing first exhibits a poorer predicted outcome than a randomly chosen pair of cases. The C-index’s interpretation as a misclassification probability renders it particularly appealing for prediction error estimation. Another advantageous aspect is its independence from a fixed single evaluation time, distinguishing it from alternative measures of survival performance. Additionally, the C-index is specifically designed to account for censoring^51^, further enhancing its utility in assessing prediction performance in survival analysis^52^. To compute the C-index, it is imperative to establish the criteria for defining a worse predicted outcome. Herein, we adopt the following approach. Consider $t_1^*, t_2^*,...,t_N^*$ as the set of all unique event times in the dataset. Individual *i* was deemed to have a worse outcome than individual *j* if (Eq. 12)

12 $$\begin{aligned} \sum _{k=1}^{N}\hat{H}_e^*(t_k^*|C_i)>\sum _{k=1}^{N}\hat{H}_e^*(t_k^*|C_j) \end{aligned}$$

The computation of the C-index involved the following steps:

- All possible pairs of cases were generated from the dataset.
- We excluded pairs where the shorter survival time was censored. Additionally, omitted pairs (*i*, *j*) if $T_i=T_j$ unless at least one of them corresponds to HIV infection. Let the total number of permissible pairs be referred to as *Permissible*.
- For each permissible pair:
  - If $T_i\ne T_j$ a count of 1 was assigned if the shorter survival time exhibited a worse predicted outcome, and 0.5 was assigned if the predicted outcomes were tied.
  - If $T_i=T_j$ and both were HIV infections, a count of 1 was assigned if the predicted outcomes were tied otherwise a value of 0.5 was assigned.
  - If $T_i=T_j$ but only one was HIV infection, a count of 1 was assigned if the event had a worse predicted outcome; otherwise, 0.5 was assigned.
  Let *Concordance* denote the resulting count over all permissible pairs.
- The C-index was computed as $C^* = \frac{Concordance}{Permissible}$
- The error rate is expressed as $Error=1-C^*$ where $0\le Error \le 1$. A value of 0.5 indicates that a procedure performs no better than random guessing, while a value of 1 signifies perfect accuracy.

#### Integrated Brier score (IBS)

in evaluating and contrasting the predictive accuracy of all models in this study, we employed the integrated Brier scores (IBS) measure^53^. The IBS reflects the mean squared variation between the observed survival status and the survival probability predicted at a given time *t*. It is worth noting that the IBS ranges from 0 to 1, with 0 signifying the optimal IBS value. To compute the Brier scores (BS) measure, we utilized the test sample of size $n_{nest}$^49^ as follows (Eq. 13);

13 $$\begin{aligned} BS(t)=\frac{1}{n_{nest}}\sum _{i=1}^{n_{nest}}\left\{ \left[ 0-\hat{S}(t|C)\right] ^2\frac{I(t_i\le t,\theta _i=1)}{\hat{G}(t_i|C)}+\left[ 1-\hat{S}(t|C)\right] ^2\frac{I(t_i>t)}{\hat{G}(t|C)}\right\} \end{aligned}$$

where $\hat{G}(t|C)\approx P(C^*>t|C=c)$ represents the Kaplan-Meier estimate for the conditional survival function of the number of censoring times, $C^*$ is the c-index and *C* is the set of covariates (cytokines and baseline variables). Consequently, the integrated Brier score (IBS) was computed as follows (Eq. 14);

14 $$\begin{aligned} IBS=\int _{0}^{max(t)}BS(t)dt \end{aligned}$$

#### Variable importance (VIMP)

The RSF offers a fully non-parametric approach to assess variable importance (VIMP)^54^. To determine the VIMP of cytokine/baseline variable $C_j$ in our dataset, the following steps were undertaken:

- For every forest tree:
  - The prediction error in the OOB data, denoted as $errOOB_b$ (using metrics such as Brier score or C-index), was computed.
  - The variable $C_j$ in the $OOB_b$ data was permuted.
  - We calculated $$\widetilde{OOB_b^j}$$
- Computed VIMP as shown in Eq. 15
  15 $$\begin{aligned} VIMP(C_j)=\frac{1}{B}\sum _{B}^{b=1}\left( \widetilde{OOB_b^j}-errOOB_b\right) \end{aligned}$$

High-importance values signify variables with predictive capacity, while zero or negative values denote non-predictive variables that may be filtered out^40^.

#### SHapley additive explanations (SHAP) values

SHAP values offers a robust way to explicate the outcomes of machine learning models^55^. Utilizing a game-theoretic framework, it evaluates contribution of each feature to the ultimate prediction^56^. In the game-theoretic approach each player’s contribution to the ultimate outcome is assessed^57^. Assuming *f*(*x*) is a predictive model for response value *y* with features $x\in {\mathbb {R}}^M$, from cooperative game theory^58^, the amount that player *i* receives is defined by the following (Eq. 16)

16 $$\begin{aligned} \Phi _i(v)=\Phi _i=\sum _{S\subseteq N\left\{ i\right\} }\frac{|S|!(M-|S|-1)!}{M!}\left( v(S\cup \left\{ i\right\} )-v(S)\right) \end{aligned}$$

with a conditional expectation of (Eq. 17)

17 $$\begin{aligned} v_{KerSHAP}(S)=\frac{1}{K}\sum _{k=1}^{K}f\left( x_{{\bar{S}}}^k, x_S^*\right) \end{aligned}$$

Where $\Phi _i(v)$ is the SHAP value for the feature *i*, *M* is a set of all features, *v*(*S*) is the trained model on the subset of feature *S* and $v(S\cup \left\{ i\right\}$ is the restricted input of *v* given subset of features *S* and *i*. Within machine learning, each feature is allocated an importance value indicating its impact on the model’s output. Features exhibiting positive SHAP values contribute positively to the prediction, whereas those with negative values exert a negative influence. The magnitude of these values serves as a gauge of the strength of their respective effects^56^.

#### Ablation studies

Ablation studies can be an effective way to investigate the impact of individual features or groups of features on the model’s predictive performance^59^. We assessed the contribution of features to HIV risk by analyzing changes in the VIMP measure as features are progressively added or removed from the model. The study proceeded as follows:

- We started with all available features and compute the VIMP for each, ranking them by importance. We then calculated the C-index to assess the overall performance.
- Selected the top n features based on their VIMP rankings. Fitted the RSF model with only these top n features, calculated performance metrics (C-index), and recorded the overall model performance.
- Progressively we added the next n highest-ranked features to the RSF model, recalculating the performance metric at each step. For each addition, we observed the marginal improvement (or deterioration) in the model’s performance.
- The process of adding features continued until we observed that the marginal improvement becomes negligible.
- We identified n_max which represents the maximum number of features needed to reach an optimal balance between model complexity and performance, after which adding features does not yield meaningful predictive gains.

## Results

### Survival support vector machine analysis

We fitted two SSVM models, one based on the mean of individual cytokine covariate measurements and the other based on the difference between the last and first recorded cytokine measurement. Each model incorporated 48 cytokine and 25 baseline variables as covariates, with their characteristics summarized in Table 1. Using the *survivalsvm* package in R, which offers flexible modeling options (e.g., regression, ranking, and hybrid methods), we specifically employed the hybrid SVM approach for its ability to combine elements of regression and ranking, as outlined in Eq. (2). The *gamma*.*mu* parameter was set to 0.5 to balance regularization, while *opt*.*meth* was set to *quadprog*, invoking the *quadprog* package for quadratic programming. Additionally, the additive kernel ($add\_kernel$) was applied to map input data into a higher-dimensional space, enhancing class separation. To address differentiation, we used the *diff*1 method, which avoids the assumption of an uncensored first data point. Model performance, measured by the C-index (Table 2), indicated that the difference-based model outperformed the mean-based model, evidenced by a higher C-index. The analysis was performed with a fixed seed (*set*.*seed*(32024)) to ensure reproducibility and 7 CPU cores to optimize computational efficiency within an R environment.

**Table 1:** SSVM results for mean and difference models.

	Mean model (N=560)*	Difference model (N=560)*
Survival SVM approach	Hybrid	Hybrid
Type of kernel	add_kernel	add_kernel
Method use to build 1NN difference	diff1	diff1
Optimization solver used	quadprog	quadprog
Number of support vectors retained	556	555
survivalsvm version	0.0.5	0.0.5

*Analysis performed using the 80% training data set.

**Table 2:** SSVM performance results for mean and difference models.

Model	C-index (SD)
Mean model (N=139)*	0.6962 (0.0392)
Difference model (N=139)*	0.7180 (0.4361)

*Analysis performed using the 20% testing data set.

### Random survival forest analysis

We fitted two random survival forest models one based on the mean of the cytokines and the other on the difference of the cytokines, incorporating survival trees constructed with both log-rank and log-rank score split rules applied to the datasets. The covariates for these models consisted of 48 cytokines and 25 baseline variables. The summarized characteristics of these fitted models are presented in Table 3. To ensure reproducibility, a fixed seed (*set*.*seed*(32024)) was applied. We specified 500 trees ($ntree = 500$) to achieve robust ensemble learning, while the minimum terminal node size ($nodesize = 50$) was set to control tree depth and prevent overfitting. Additionally, we set 5 random splits per node ($nsplit = 5$) to enhance predictive performance through variability in split candidates. The *block*.*size* was set to 1 allowing the model to build one tree per computational block, and feature importance ($importance = TRUE$) was enabled to highlight influential predictors. These RSF models were executed in R using the *randomForestSRC* package with 7 CPU cores for parallel computing to optimize computational efficiency.

**Table 3:** RSF results for mean and difference models using log-rank and log-rank score split rule.

	Mean model (N=560)*		Difference model (N=560)*	
	Log-rank	Log-rank score	Log-rank	Log-rank score
Number of HIV Infection	64	64	64	64
Number of trees	500	500	500	500
Forest terminal node size	50	50	50	50
Average no. of terminal nodes	8.276	8.184	8.376	8.206
No. of variables tried at each split	9	9	9	9
Total no. of variables	73	73	73	73
Resampling used to grow trees	swor	swor	swor	swor
Resample size used to grow trees	354	354	354	354
Analysis	RSF	RSF	RSF	RSF
Family	surv	surv	surv	surv
Splitting rule	log-rank	log-rank score	log-rank	log-rank score
Number of random split points	5	5	5	5
Error rate	31.10%	35.72%	27.71%	34.85%

*Analysis performed using the 80% training data set.

**Fig. 1:**
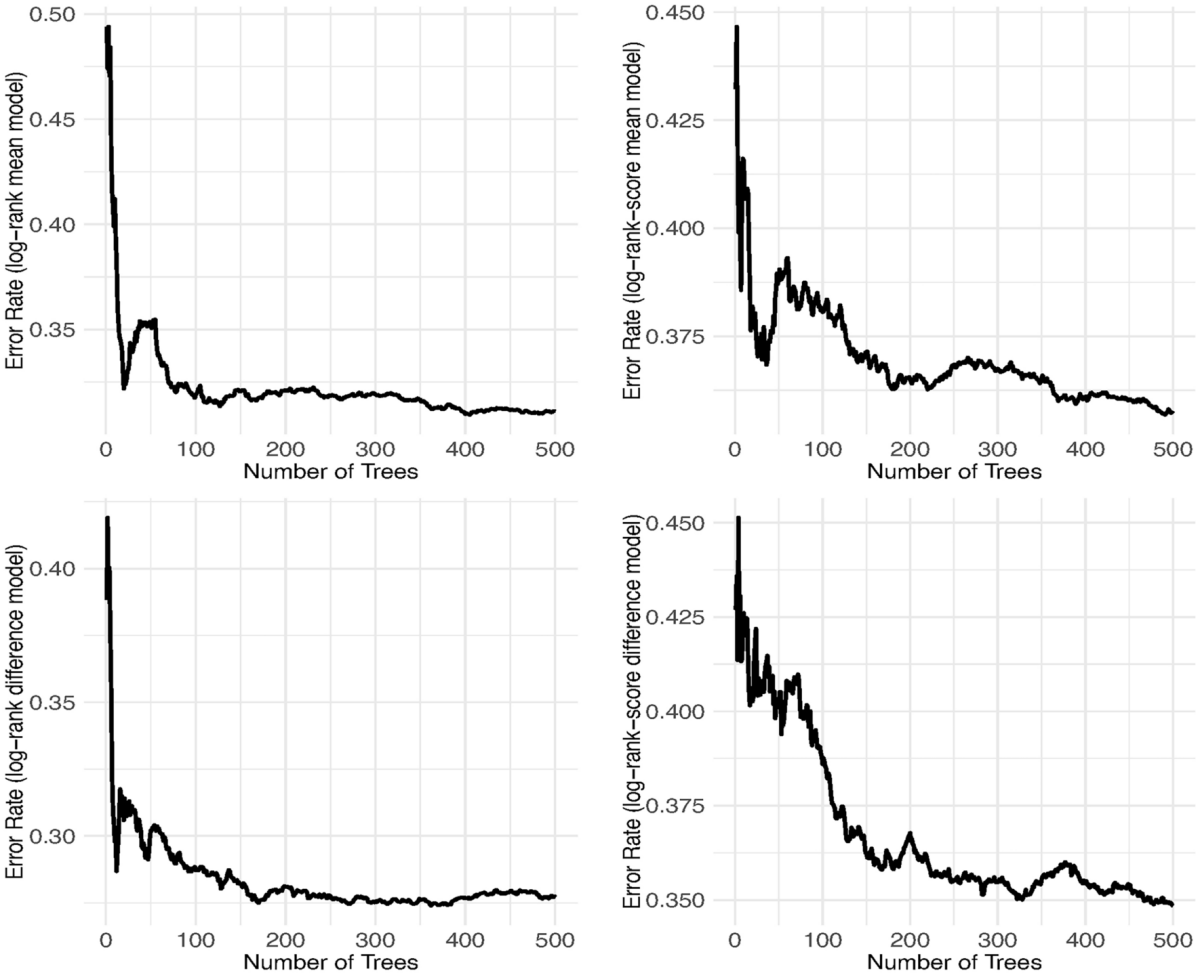
The prediction error for the survival forests of 500 trees for mean (upper panels) and difference models (lower panels) for the log-rank and log-rank score in the left and right panels respectively using 80% training dataset.

The random survival forest based on the mean cytokine covariate model was fitted, yielding error rates of 31.10% and 35.72% for forests constructed using survival trees based on the log-rank and log-rank score split rules, respectively. Notably, these error rates for the mean model are considerably greater than those of the difference model, with error rates of 27.71% and 34.85%, respectively (as detailed in Table 3). This observation suggests the superior performance of the difference model over the mean model with the difference model using the log-rank split rule achieving the best performance among all models. Moreover, the error rate from the log-rank split rule was lower than that from the log-rank score split rule for both derived cytokine covariate models. Additionally, we conducted further analysis by fitting the models with varying numbers of survival trees (100, 200, 300, 400 and 500). The error rates stabilized for the log-rank split rule from 200 survival trees, whereas the log-rank score split rules had not yet stabilized even at 500 survival trees for both models, as illustrated in Fig. 1.

**Fig. 2:**
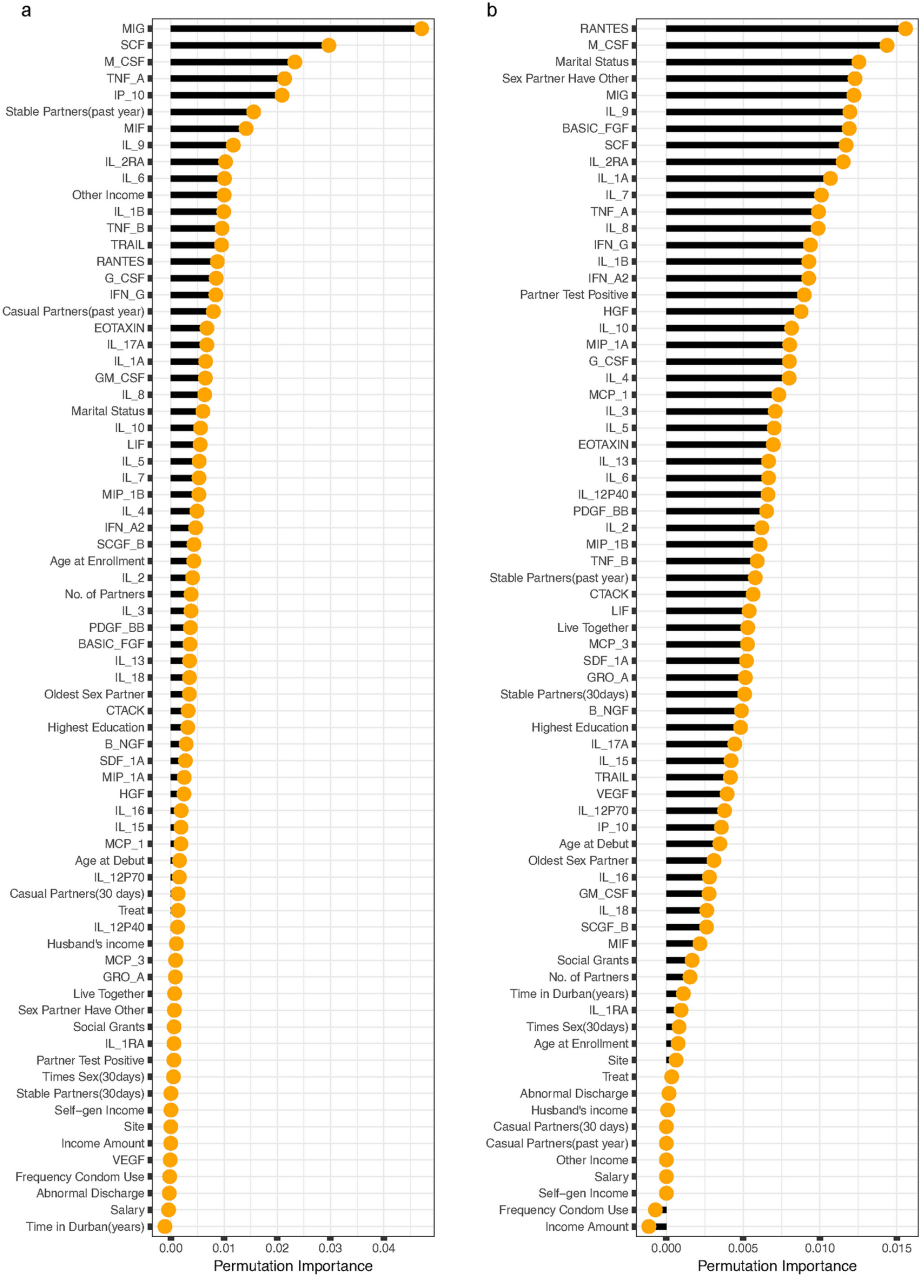
The rank of the most predictive cytokines and baseline variables for HIV incidence among women aged 18-40 years for the mean model. The variable importance is determined separately for the left and right panels using the log-rank (**a**) and log-rank score (**b**) split rules, respectively.

The permutation importance measure was employed to ascertain the most important cytokines and baseline variables linked to HIV incidence^47^. The utilization of RSF allows the inclusion of all 48 cytokines and 25 baseline covariates, regardless of their conformity with the Cox PH assumption because the assumption is not a prerequisite. RSF operates purely on a non-parametric basis, thereby obviating the need for adherence to the Cox PH assumption during the covariate selection process^60^. In the RSF analysis of the mean model (refer to Fig. 2), the top 20 cytokines and baseline covariates most crucial and strongly linked with HIV incidence, as identified using the log-rank split rule, were: MIG, SCF, M-CSF, TNF-A, IP-10, number of stable partners in the past year, MIF, IL-9, IL-2RA, IL-6, other sources of income, IL-1B, TNF-B, TRAIL, RANTES, G-CSF, IFN-G, number of casual partners in the past year, EOTAXIN and IL-17A. While the top 20 identified by log-rank score were: RANTES, M-CSF, marital status, sex partner have other partner, MIG, IL-9, BASIC-FGF, SCF, IL-2RA, IL-1A, IL-7, TNF-A, IL-8, IFN-G, partner’s HIV status, HGF, IL-10 and MIP-1A.

**Fig. 3:**
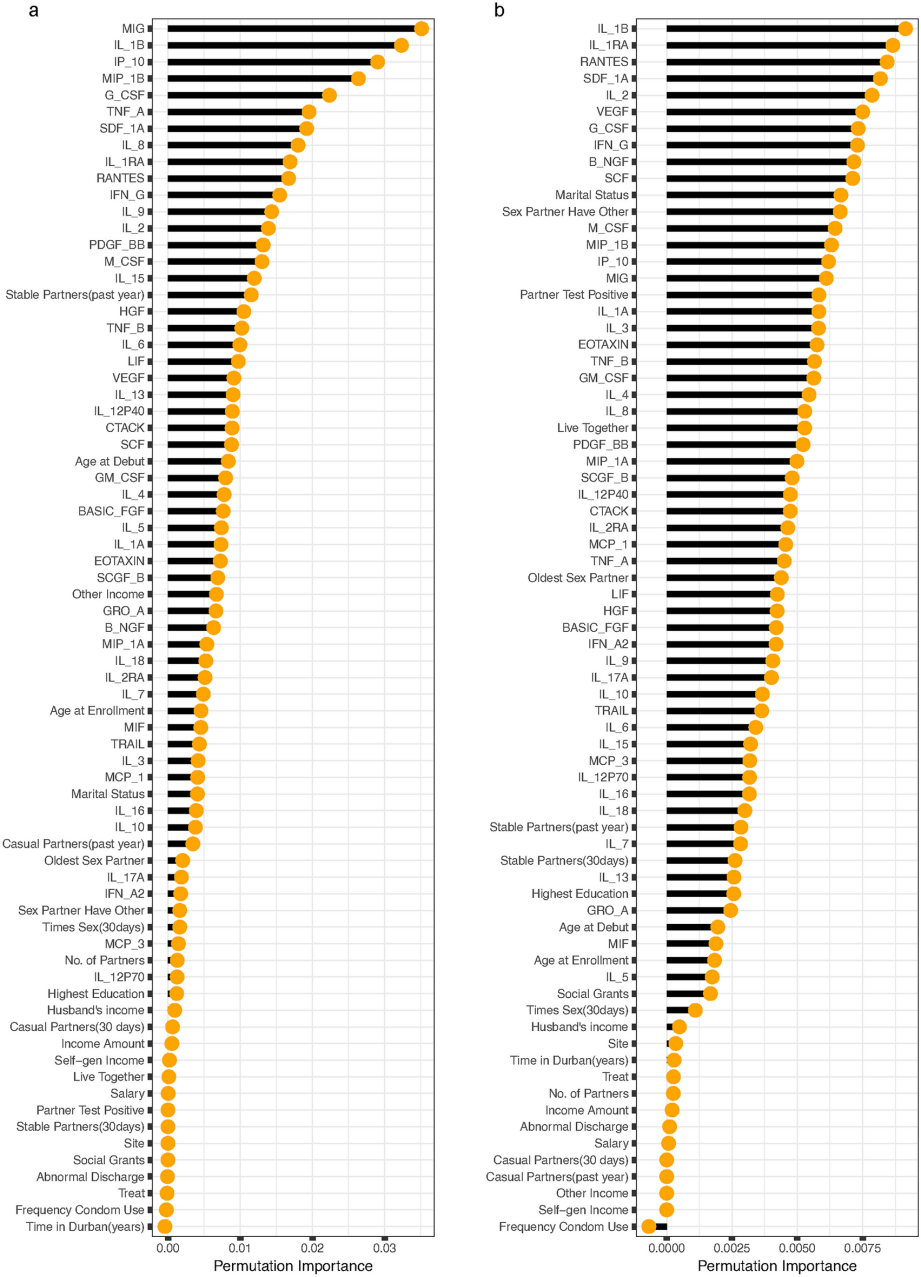
The rank of the most predictive cytokines and baseline variables for HIV incidence among women aged 18–40 years for the difference model. The variable importance is determined separately for the left and right panels using the log-rank (**a**) and log-rank score (**b**) split rules, respectively.

According to RSF analysis of the difference mo-del (refer to Fig. 3), the top 20 cytokines and baseline covariates most crucial and strongly linked with HIV incidence, identified using the log-rank split rule, were MIG, IL-1B, IP-10, MIP-1B, G-CSF, TNF-A, SDF-1A, IL-8, IL-1RA, RANTES, IFN-G, IL-9, IL-2, PDF-BB, M-CSF, IL-15, number of stable partners in the past year, HGF, TNF-B and IL-6. While the top 20 identified by log-rank score were IL-1B, IL-1RA, RANTES, SDF-1A, IL-2, VEGF, G-CSF, IFN-G, B-NGF, SCF, marital status, sex partner have other partner, M-CSF, MIP-B, IP-10, MIG, partner’s HIV status, IL-1A, IL-3 and EOTAXIN.

#### Predictive performance

**Fig. 4:**
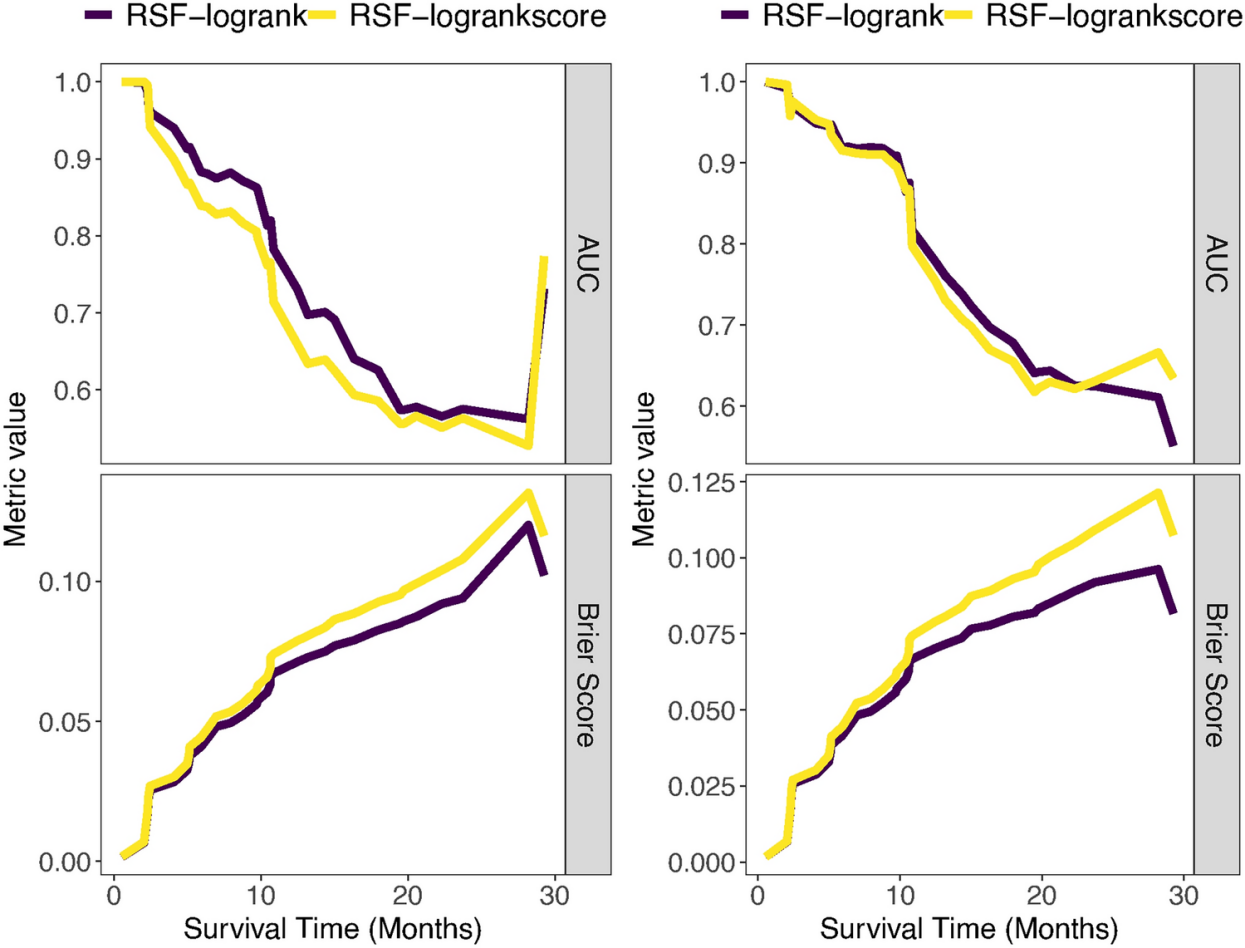
Performance comparison of the RSF models (the mean left panel and the difference models right panel) using log-rank and log-rank score split rule using performance metric AUC (top panel) and Brier score (lower panel) over time using the 80% training dataset.

**Fig. 5:**
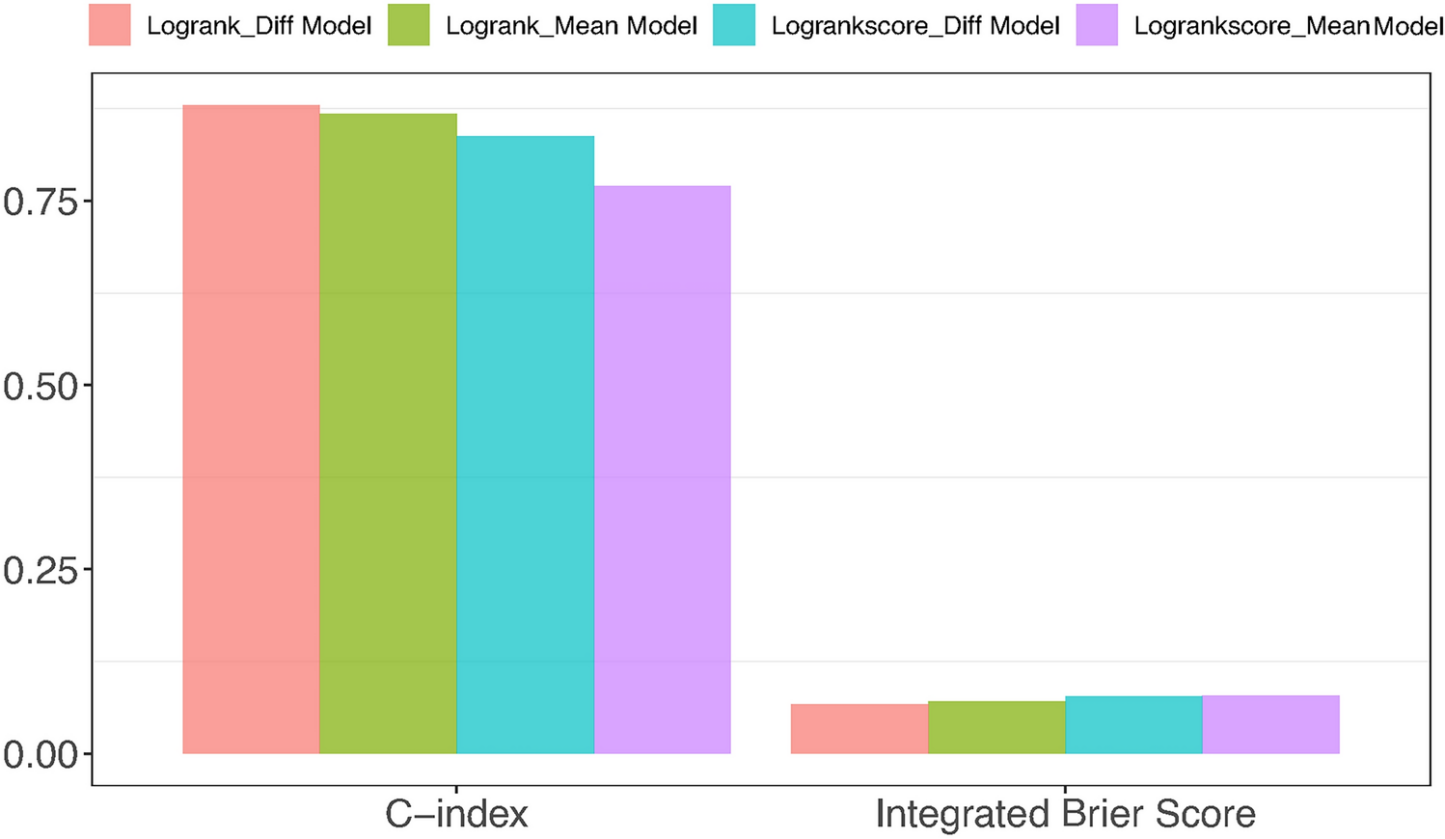
Performance comparison of the RSF models across entire time domain using the 80% training dataset.

We evaluated the model’s performance over time utilizing the performance metrics AUC and Brier scores (depicted in Fig. 4) and the overall performance of the model throughout the entire duration using the concordance index and integrated Brier scores (illustrated in Fig. 5). These evaluations were conducted for both log-rank and log-rank score split rule using the *R* package *survex*^61^. The model exhibiting lower Integrated/Brier scores and higher AUC and concordance index values was deemed to perform better over time and across the entire time domain respectively. Figure 5 indicates that the log-rank split rule of the difference model outperformed all the other models while the log-rank score split rule of the mean model performed the poorest. In general, RSF with the log-rank split rule demonstrated superior performance compared to RSF with the log-rank score split rule.

We extended our analysis using the Shapley additive explanations (SHAP) method to ascertain the relative importance of our cytokine and baseline variables. A SHAP value of 0 indicated a negligible influence of the cytokine on the prediction of HIV incidence, as depicted in Table 4. The results from the RSF mean model using the log-rank split rule indicated the following cytokines BASIC-FGF, EOTAXIN, G-CSF, IL-15, IL-4, IL-5, IL-6, IL-9, IP-10, RANTES, TNF-A, M-CSF, MIG, and SCF and the baseline variables; treatment, highest education, other source of income, years lived in Durban, age at debut, number of stable partners in the past year, oldest partner and frequency of condom use had a positive influence to the prediction of HIV incidences. Moreover, the cytokines GM-CSF, IL-15, IL-17A, IL-2, IL-18, MIF, TNF-B, TRAIL and B-NGF and the baseline variables; number of partners and abnormal discharge had a negative influence on the prediction of HIV infections.

**Table 4:** RSF average SHAP values for the mean and the difference models using log-rank and log-rank score split rule.

Covariates	Mean Model (N=139)*		Difference model (N=139)*	
	log-rank	log-rank score	log-rank	log-rank score
Treatment	0.0002	0.0000	0.0001	0.0002
Site	0.0000	0.0000	0.0001	0.0001
Live with partner	0.0000	0.0002	0.0001	0.0003
Highest education	0.0001	0.0000	0.0001	0.0001
Self Gen Income	0.0000	0.0000	0.0001	0.0001
Salary	0.0000	0.0000	0.0001	0.0001
Husband Income	0.0000	0.0000	0.0001	0.0001
Social grants	0.0000	0.0000	0.0001	0.0001
Other income	0.0003	0.0000	0.0002	0.0001
Income amount	0.0000	0.0000	0.0001	0.0001
Time in durban (years)	0.0001	0.0001	0.0000	0.0002
Age at enrollment	0.0000	0.0000	0.0001	0.0001
Marital status	0.0000	$-$0.0001	0.0000	$-$0.0001
Age at debut	0.0001	0.0000	0.0002	0.0001
Total partners	$-$0.0001	0.0000	0.0001	0.0001
No. stable partners (past year)	0.0003	0.0000	0.0002	0.0000
No. casual partners (past year)	0.0000	0.0000	0.0001	0.0001
No. stable partners (30 Days)	0.0000	0.0000	0.0001	0.0001
No. casual partners (30 Days)	0.0000	0.0000	0.0000	0.0001
No. sexual intercourse (30 Days)	0.0000	0.0000	0.0001	0.0001
Oldest partner	0.0001	0.0000	0.0001	0.0001
Sex partner have other	0.0000	0.0000	0.0000	0.0001
Condom use	0.0001	0.0001	0.0001	0.0002
Abnormal Discharge	$-$0.0001	0.0000	0.0000	0.0001
Sex Partner’s HIV status	0.0000	0.0003	0.0001	0.0004
BASIC_FGF	0.0001	0.0001	0.0003	$-$0.0005
EOTAXIN	0.0001	0.0000	0.0014	0.0003
G_CSF	0.0003	0.0000	$-$0.0026	$-$0.0017
GM_CSF	$-$0.0001	0.0000	$-$0.0039	$-$0.0005
IFN_G	0.0000	0.0001	$-$0.0005	$-$0.0005
IL_10	0.0000	0.0000	0.0001	$-$0.0002
IL_12P70	0.0000	0.0000	0.0000	$-$0.0008
IL_13	0.0000	0.0001	$-$0.0019	$-$0.0008
IL_15	$-$0.0001	0.0000	$-$0.0017	$-$0.0003
IL_17A	$-$0.0001	0.0000	0.0001	$-$0.0006
IL_1B	0.0001	0.0000	$-$0.0047	$-$0.0013
IL_1RA	0.0000	0.0000	$-$0.0053	$-$0.0011
IL_2	$-$0.0001	0.0000	$-$0.0029	$-$0.0010
IL_4	0.0001	0.0000	0.0000	$-$0.0001
IL_5	0.0005	0.0002	0.0006	0.0004
IL_6	0.0005	0.0001	$-$0.0013	$-$0.0012
IL_7	0.0000	0.0001	0.0002	$-$0.0005
IL_8	0.0001	0.0000	$-$0.0029	$-$0.0010
IL_9	0.0001	$-$0.0001	0.0000	$-$0.0002
IP_10	0.0001	0.0000	$-$0.0018	$-$0.0009
MCP_1	0.0000	0.0000	$-$0.0025	$-$0.0011
MIP_1A	0.0000	0.0001	0.0004	$-$0.0004
MIP_1B	0.0000	0.0000	$-$0.0054	$-$0.0014
PDGF_BB	0.0000	0.0000	$-$0.0026	$-$0.0009
RANTES	0.0001	0.0002	$-$0.0036	$-$0.0018
TNF_A	0.0003	0.0001	0.0010	0.0000
VEGF	0.0000	0.0001	$-$0.0035	$-$0.0013
GRO_A	0.0000	0.0000	$-$0.0011	$-$0.0007
HGF	0.0000	$-$0.0001	$-$0.0021	$-$0.0011
IFN_A2	0.0000	0.0000	$-$0.0005	$-$0.0002
IL_12P40	0.0000	0.0001	$-$0.0039	$-$0.0005
IL_16	0.0000	$-$0.0001	$-$0.0001	$-$0.0005
IL_18	$-$0.0002	0.0000	$-$0.0001	0.0001
IL_1A	0.0000	0.0001	$-$0.0005	$-$0.0009
IL_2RA	0.0000	0.0000	$-$0.0005	$-$0.0008
IL_3	0.0000	0.0000	$-$0.0002	$-$0.0004
LIF	0.0000	0.0000	0.0000	$-$0.0005
M_CSF	0.0011	0.0003	$-$0.0013	$-$0.0011
MCP_3	0.0000	$-$0.0001	0.0002	0.0002
MIF	$-$0.0005	0.0000	$-$0.0010	$-$0.0009
MIG	0.0004	$-$0.0001	$-$0.0035	$-$0.0008
SCF	0.0002	0.0000	$-$0.0008	$-$0.0008
SDF_1A	0.0000	0.0000	$-$0.0068	$-$0.0013
TNF_B	$-$0.0002	0.0000	0.0005	$-$0.0001
TRAIL	$-$0.0002	0.0000	$-$0.0001	$-$0.0006
B_NGF	$-$0.0001	0.0000	0.0007	0.0001

*Analysis performed using the 20% testing data set.

Considering the results from the RSF mean model using the log-rank score split rule, the cytokines that had a positive effect on the prediction of HIV infections were BASIC-FGF, IFN-G, IL-13, IL-5, IL-6, IL-7, MIP-1A, RANTES, TNF-A, VEGF, IL-12P40, IL_1A, and M-CSF. Moreover, those that negatively influenced the prediction of HIV incidence were IL-9, HGF, IL-16, MCP-3 and MIG. Additionally, the baseline variables site, highest education, self generating income, salary, husband’s income, social grant, other income, income amount, age at debut, number of partners, number of stable partner in the past year, number of casual partner in the past year or 30 days, oldest partner, sex partner have other partner and abnormal discharge had no effect on the prediction of HIV incidence while living with a partner, years lived in Durban, frequency of condom use and sex partner’s HIV status positively influence the prediction of HIV infection. Only marital status negatively impacted the prediction of HIV incidence.

The results of the RSF difference model using the log-rank split rule showed that the cytokines BASIC-FGF, EOTAXIN, IL-10, IL-17A, IL-5, IL-7, MIP-1A, TNF-A, MCP-3, TNF-B and B-NGF positively impacted the prediction of HIV incidence. Furthermore, G-CSF, GM-CSF, IFN-G, IL-13, IL-15, IL-1B, IL-1RA, IL-2, IL-6, IL-8, IP-10, MCP-1, MIP-1B, PDGF-BB, RANTES, GRO-A, VEGF, HGF, IFN-A2, IL-12P40, IL-16, IL-18, IL-1A, IL-2RA, IL-3, MCS-F, MIF, MIG, SCF, SDF-1A and TRAIL negatively influenced the prediction of HIV infections. The baseline covariates that positively influenced the prediction of HIV incidence were; treatment, site, living with partner, highest education, self-generating income, salary, husband’s income, social grant, other income, income amount, age at enrollment, age at debut, number of partners, number of stable partners in the past year, number of casual partners in the past year and past 30 days, the number of sexual intercourse, oldest partner, frequency of condom use and sex partner’s HIV status.

The results of the RSF difference model using the log-rank score split rule showed that the cytokine EOTAXIN, IL-5, IL-18, MCP_3, and B-NGF positively impacted the prediction of HIV incidence. However, G-CSF, GM-CSF, IFN-G, IL-10, BASIc-FGF, IL-12P70, IL-13, IL-15, IL17-A, IL-1RA, IL-2, IL-4, IL-6, IL-7, IL-8, IL-9, IP-10, MCP_1, MIP-1A, MIP-1B, PDGF-BB, RANTES, VEGF, GRO-A, VEGF, HGF, IFN-A2, IL-12P40, IL-16, IL-1A, IL-2RA, IL-3, LIF, MIF, MIG, SCF, SDF-1A, TNF-B and TRAIL negatively influenced the prediction of HIV infections. The baseline variables that positively influenced the prediction of HIV incidence were; treatment, site, living with partner, highest education, self-generating income, salary, social grant, other income, income amount, years lived in Durban, age at debut, number of partners, number of stable partners in the past 30 days, number of casual partners in the past year or 30 days, number of sexual intercourse, oldest partner, sex partner have other partner, frequency of condom use, abnormal discharge and sex partner’s HIV status. Marital status had a negative influence on the prediction of HIV incidence.

**Fig. 6:**
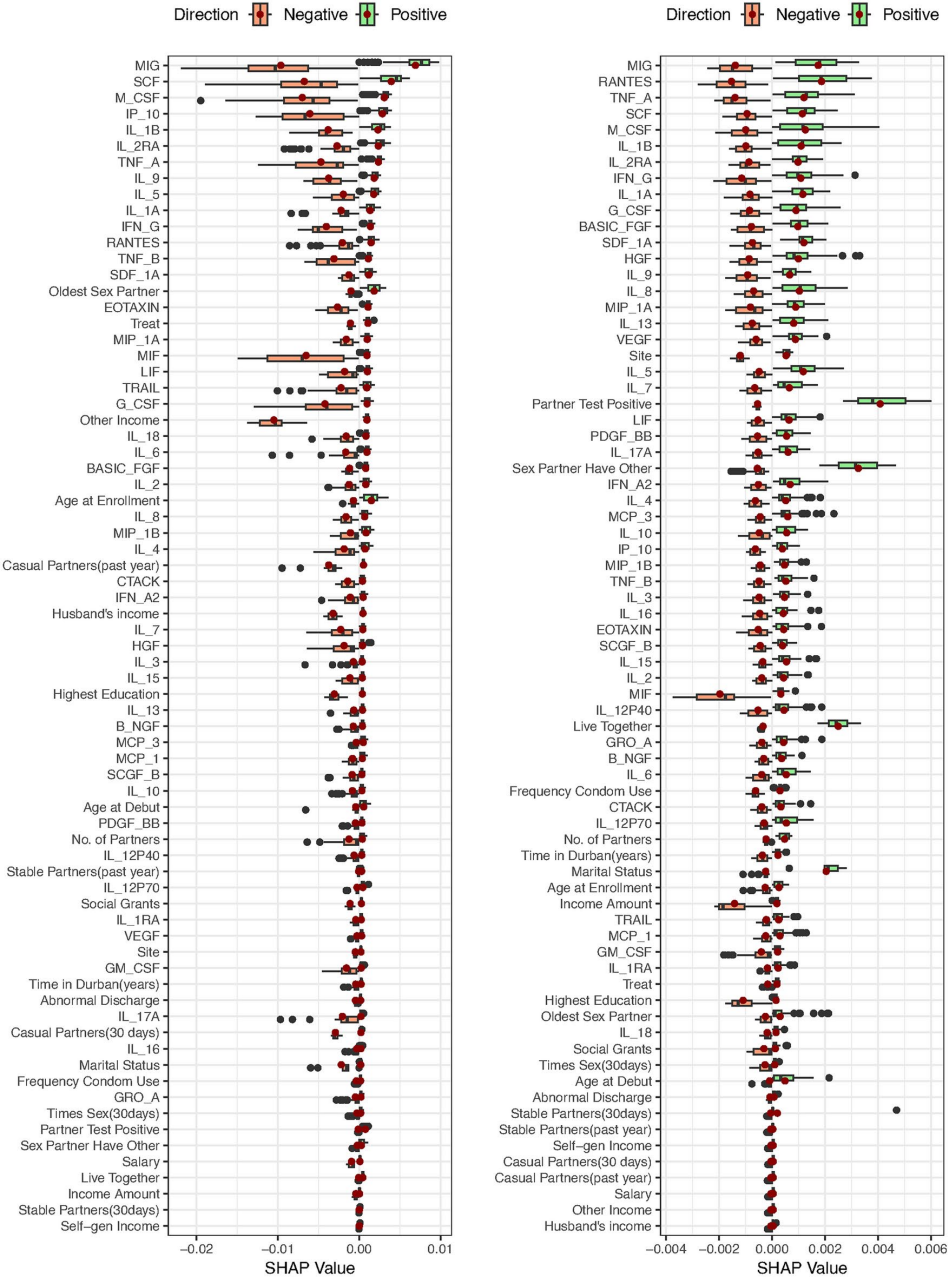
Predictive performance of the RSF mean model for log-rank and log-rank score in the left and right panel respectively, using 20% training dataset.

**Fig. 7:**
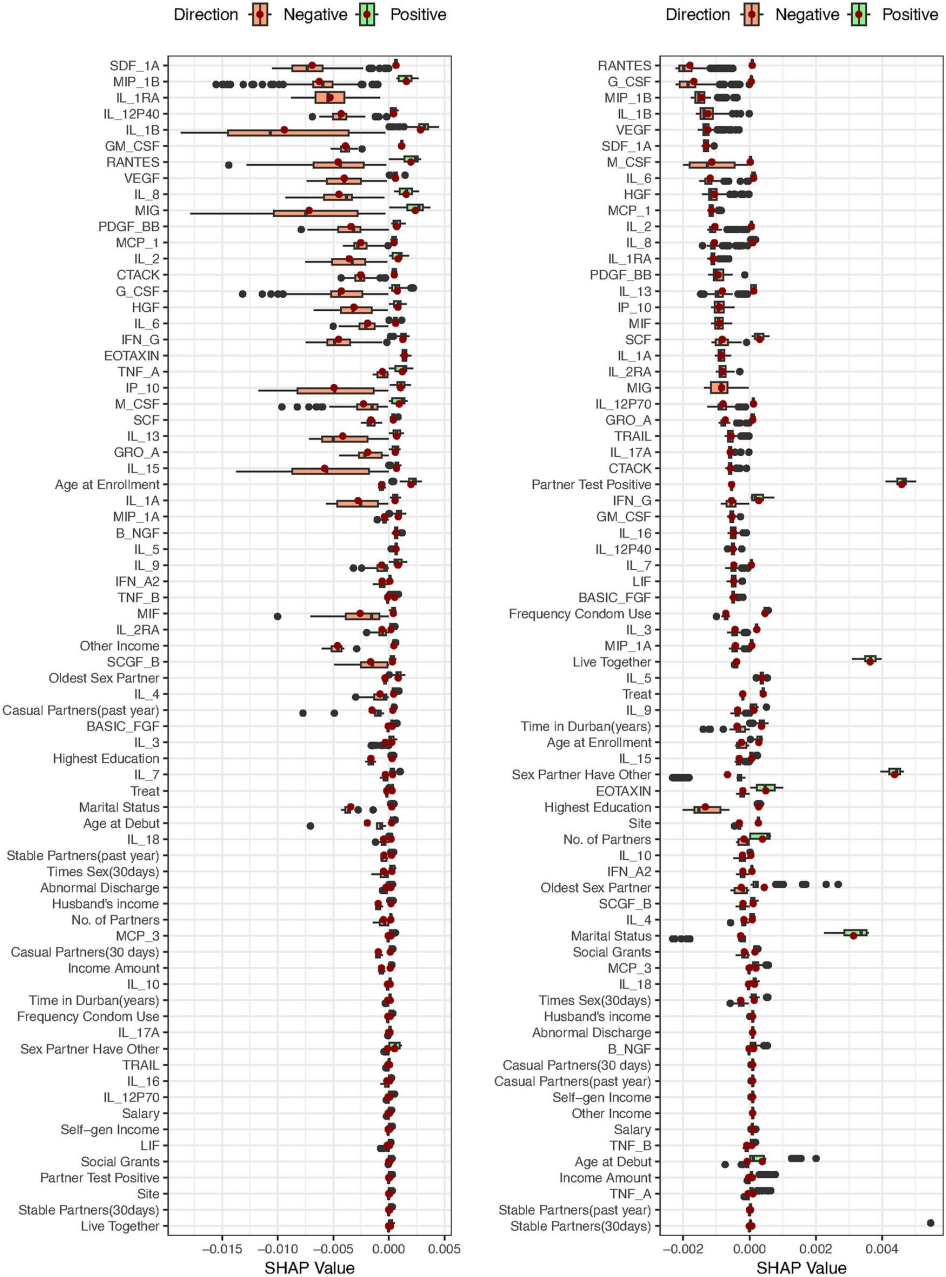
Predictive performance of the RSF difference model for log-rank and log-rank score in the left and right panel respectively, using 20% training dataset.

Figures 6 and 7 depict the prediction strength of the variable effect ranked from the highest to the lowest. The vertical axis presents the cytokine and baseline variable names ordered by importance, with higher-ranked variables appearing at the top and the SHAP values on the horizontal axis. Cytokines/baseline variables that elevated the predictions are depicted in green, while those that decreased the predictions are shaded in light red. Each data point represents a row from the original dataset, with dark red points denoting the mean SHAP value. The majority of cytokines exhibited a greater influence on the prediction of HIV incidence, as evidenced by their higher rankings compared to baseline variables in both the mean and difference models when employing the log-rank and log-rank-split rules. Remarkably, numerous cytokine and baseline variables in both the mean and difference models, utilizing both log-rank and log-rank score split rules, positively influenced the prediction of HIV incidence.

The top twenty variables with the most significant impact on the prediction of HIV incidence identified by the SHAP values of the RSF mean model when employing the log-rank split rule included; MIG, SCF, M-CSF, IP-10, IL-1B, IL-2RA, TNF-A, IL-9, IL-5, IL-1A, IFN-G, RANTES, TNF-B, SDF-1A, oldest partner, EOTAXIN, treatment, MIP-1A, MIF and LIF while those identified when utilizing log-rank score split rule were; MIG, RANTES, TNF-A, SCF, M-CSF, IL-1B, IL-2RA, IFN-G, IL-1A, G-CSF, SASIC-FGF, SDF-1A, HGF, IL-9, IL-8, MIP-1A, IL-13, VEGF, and IL-5. The top twenty variables with the most significant impact on the prediction of HIV incidence identified by the SHAP values of the RSF difference model when employing the log-rank split rule included; SDF-1A, MIP-1B, IL-1RA, IL-12P40, IL-1B, GM-CSF, RANTES, VEGF, IL-8, MIG, PDGF-BB, MCP-1, IL-2, CTACK, G-CSF, HGF, IL-6, IFN-G, EOTAXIN and TNF-A. Those identified by the SHAP values of the RSF difference model utilizing the log-rank score split rule were RANTES, G-CSF, MIP-1B, IL-1B, VEGF, SDF-1A, M-CSF, IL-6, HGF, MCP-1, IL-2, IL-8, IL-1RA, PDGF-BB, IL-13, IP-10, MIF, SCF, IL-1A and IL-2RA.

**Fig. 8:**
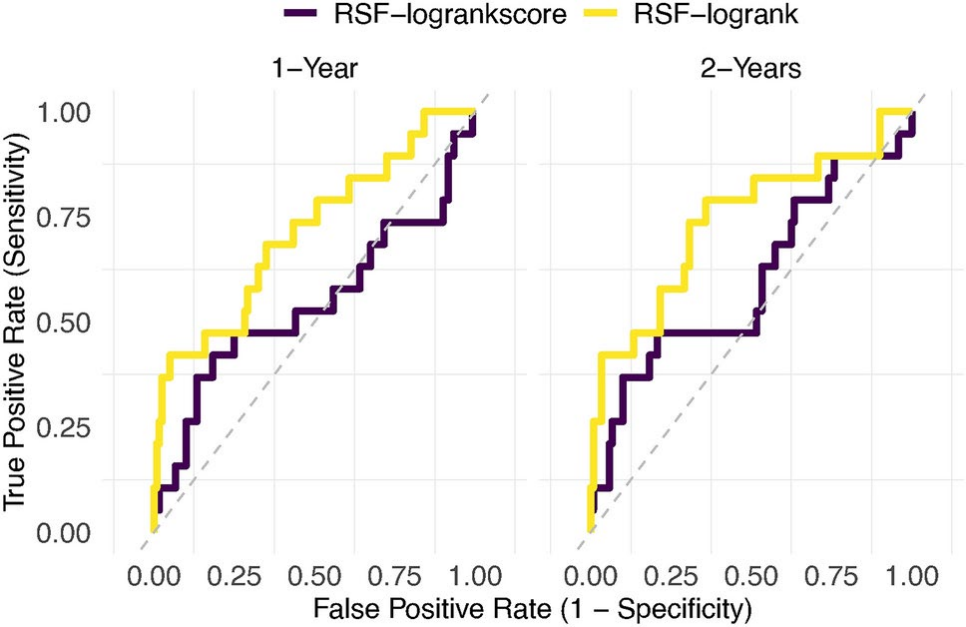
ROC curves of the RSF mean model for log-rank and log-rank score in the left and right panel respectively, using 20% training dataset.

**Fig. 9:**
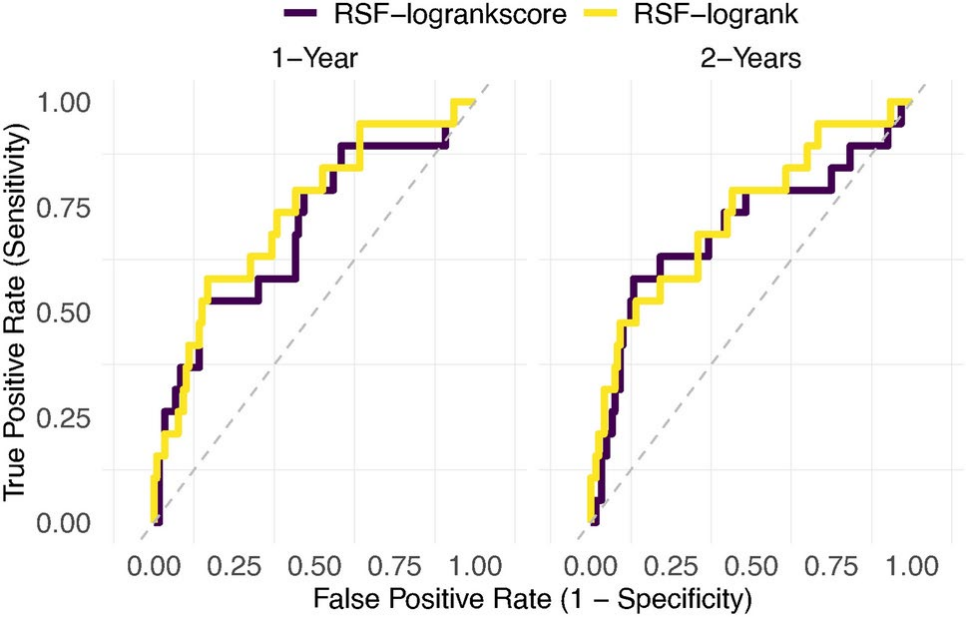
ROC curves of the RSF difference model for log-rank and log-rank score in the left and right panel respectively, using 20% training dataset.

Furthermore, ROC curves were plotted, as illustrated in Figs. 8 and 9, to assess the performance of the mean and difference models in predicting HIV incidence using the log-rank and log-rank score splitting rules. These curves compare the true positive rate (sensitivity) with the false positive rate (1 - specificity), providing an essential means for evaluating the predictive accuracy of RSF models^62^. Overall the log-rank split rule had a better predictive performance over one and two years than the log-rank score for the mean and difference models.

**Table 5:** Comparison of the models’ predictive performance using the C-index.

Models	C-index
RSF mean model (log-rank)	0.8676
RSF mean model (log-rank score)	0.7697
RSF difference model (log-rank)	0.8801
RSF difference model (log-rank score)	0.8380
SSVM mean model	0.6962
SSVM difference model	0.7180

#### Ablation studies analysis

The ablation study presented in Table 6 illustrates the impact of feature set size on model performance, evaluated through the RSFs models using the log-rank and log-rank score split rules both the mean and difference models, each trained on 80% of the dataset (N=560). For the mean model, the inclusion of the top 50 and 60 features yielded the highest C-index of 0.8704 and 0.7716 using the log-rank and log-rank score split rules respectively, indicating that this feature subsets provided the optimal predictive balance. The model’s performance slightly declined when all features were included, with a C-index of 0.8676 (log-rank) and 0.7697 (log-rank score), suggesting decreased predictive performance when adding more features. For the difference model, the highest performance was achieved when all features were included, with the C-indices of 0.88801 and 0.8380 for the log-rank and log-rank score split rules respectively. The results indicate that the full feature set provided the optimal predictive accuracy, though using fewer features, particularly the top 40 to 70, maintained comparably high performance levels. This suggests that while the model benefits from a comprehensive feature set, the top 40-70 features capture most of the predictive value, as seen in the minimal decrease in both log-rank and log-rank score split rules when reducing from all features to 40-70 features. Overall, both models demonstrate strong performance with reduced feature sets, highlighting the importance of selecting key features. For practical purposes, selecting the top 50 features in the mean model and the top 40-70 in the difference model may provide a near-optimal balance between model complexity and predictive power.

**Table 6:** Ablation study comparing the C-indices for different feature set selected from a set ordered by VIMP.

Feature set	Mean model (N=560)*		Difference model (N=560)*	
	log-rank	log-rank score	log-rank	log-rank score
All features	0.8676	0.7697	0.8801	0.8380
Top 20 features	0.8470	0.7663	0.8677	0.8317
Top 30 features	0.8531	0.7563	0.8722	0.8228
Top 40 features	0.8562	0.7519	0.8799	0.8225
Top 50 features	0.8704	0.7617	0.8779	0.8237
Top 60 features	0.8632	0.7716	0.8777	0.8323
Top 70 features	0.8657	0.7614	0.8787	0.8378

*Analysis performed using the 80% training data set.

## Discussion

Cytokines are crucial signaling molecules involved in modulating immune responses and inflammation^63^. Aberrant cytokine profiles have been implicated in various diseases, including HIV/AIDS. Understanding the role of cytokines as predictors of HIV incidence can provide valuable insights into the immunological mechanisms underlying infection susceptibility^64^. The early stages of HIV infection involve significant inflammation and immune disruption, particularly in the gut mucosa, alongside the genitalia, which correlates with a higher plasma viral load. Inflammation plays a central role in HIV pathogenesis, with levels of inflammatory cytokines and chemokines signaling infection that draw immune cells to the mucosa, commonly used as inflammation biomarkers in the female reproductive tract^65^. Studies indicate that elevated pro-inflammatory cytokines are linked to higher HIV acquisition rates, and specific cytokine profiles can effectively predict future HIV infection. These cytokine patterns directly influence HIV disease progression, marked by a severe cytokine response during the acute infection phase^66^. During HIV infection, T-helper 1 (Th1) cytokines such as interleukin (IL)-2 and antiviral interferon (IFN)-gamma typically decrease, while T-helper 2 (Th2) cytokines, including IL-4 and IL-10, as well as pro-inflammatory cytokines (IL-1, IL-6, IL-8) and tumor necrosis factor (TNF)-alpha, increase^64^. Certain cytokines like IFN-alpha, IFN-beta, and IL-16 can suppress HIV by inhibiting its replication in T cells. In contrast, others, such as IFN-gamma, IL-4, and granulocyte-macrophage colony-stimulating factor (GM-CSF), may have dual roles in promoting or inhibiting HIV^67^.

Traditionally, the Cox PH model^16^ and other models such as lognormal and weibull^68^ has been used to analyze survival data in HIV research . However, ML models such as RSF and SSVM offer several advantages over the traditional models, making them an attractive alternative for analyzing cytokine profiles in the context of HIV incidence prediction^69^. These models are advantageous because of their capability to handle high-dimensional data, non-linear relationships between predictors, and violations of the PH assumption^70^. The models can capture complex interactions and non-linear effects, providing more accurate predictions. Additionally, the models do not rely on stringent model assumptions, making them robust to model misspecifications and suitable for analyzing diverse datasets^71^. This study aimed to explore cytokine profiles as potential predictors of HIV incidence through the application of RSF and SSVM models. Given that cytokine profiles within our dataset were time-dependent, they were integrated into the survival modeling process using derived variables obtained from longitudinal measurements. Specifically, the first derived variable was computed by averaging all longitudinal measurements, while the second variable was determined by calculating the difference between the last and first longitudinal measurements. These derived variables were combined with baseline variables to construct two SSVM and two RSF models, namely the mean model and difference model in each respectively. RSF models utilized both log-rank and log-rank score split rules. We fitted the models, to identify potential cytokine profiles predictors of HIV incidence and compared their performances. The utilization of variable importance, the C-index, integrated Brier scores, SHAP values and ROC/AUC curves enhances the accuracy and interpretability of HIV risk prediction models, providing valuable insights into the immunological dynamics of HIV infection.

One of the primary benefits of RSF models is their ability to assess variable importance. The RSF calculates the importance of each predictor based on its contribution to the model’s predictive performance^47^. Among the top 20 variables deemed significant in the mean model, several cytokines emerged consistently across both the log-rank and log-rank score split rules as highly influential cytokines associated with HIV infection. These included MIG, SCF, M-CSF, TNF-A, IL-9, IL-2RA, IL-1B, RANTES, and IFN-G. Similarly, in the difference model, a set of cytokines stood out as significant across both split rules, including MIG, IL-1B, MIP-1B, SDF-1A, IL-1RA, RANTES, IFN-G, IL-2, TNF-B and M-CSF. The most common cytokines between the mean and the different models using log-rank split rule were MIG, M-CSF, TNF-G, IP-10, IL-10, IL-6, IL-1B, TNF-B, RANTES and G-CSF. For the log-rank score split rule, the common cytokines were RANTES, M-CSF, MIG, SCF, IL-1A, IFN-G and IL-1B. Notably, the cytokine profiles MIG, IL-1B, IFN-G and RANTES emerged as particularly noteworthy, consistently identified as the most important predictors of HIV incidence across both the mean and difference models using both split rules. Certain baseline characteristics were found to positively influence HIV incidence prediction across both log-rank and log-rank score split rules. These included the number of years lived in Durban and the frequency of condom use in the RSF mean model, and factors such as treatment, site, living with a partner, highest education, income-related variables, age-related variables, number of partners, number of times had sex, and frequency of condom usage variables in the RSF difference model. Notably, the frequency of condom use emerged as the most consistent baseline characteristic identified by both the RSF mean and the RSF difference models using both split rules.

The performance of the RSF models in predicting HIV incidence was assessed by maximizing the C-index and minimizing the Integrated Brier Scores^52^. The high C-index and lower IBS values obtained from RSF models indicate their superior predictive performance over SSVMs models. The most notable finding from the RSF analysis is the superior performance of the log-rank split rule over the log-rank score split rule (Table 5) in both RSF mean and difference models. The difference model for RSF and SSVM outperformed the mean model of RSF and SSVM. The RSF mean model predominantly identified cytokine profiles that had a negligible influence on the prediction of HIV incidence, while the RSF difference model was more adept at identifying cytokine profiles with a negative influence on the prediction of HIV incidence. All the RSF models outperformed SSVM models indicating that RSF is a better alternative for high dimensional survival data sets. The mean model captured the average effect of the cytokine profiles, whereas the difference model focused on modeling the effect of changes in cytokine profiles. The difference between the last observed and the first observed cytokine measurements accounts for the time effect while the mean value of the cytokine effects conceals the changing cytokine measure over time. The discrepancies between log-rank and log-rank score split rules were more pronounced under the derived mean cytokine covariate than with the difference cytokine covariate model. It may suggest if one is using the mean cytokine covariate model then the log-rank split rule works best. While if one is using the change in cytokine measurements then either split rule looks fine.

The choice of split rules in RSF models directly influences model performance by affecting how nodes are split, and which variables are selected as primary determinants of survival outcomes. The log-rank split rule selects splits that maximize the difference in survival outcomes between groups^40^. It does this by identifying cut-points that best separate subjects with different survival probabilities, favoring variables with significant distinctions between high and low-risk groups. This split rule is particularly effective for identifying variables that strongly correlate with survival differences. It helps in modeling datasets where the survival function varies clearly between groups^47^, making it well-suited for capturing global survival trends and often leading to good predictive performance. The log-rank split rule was chosen due to its robustness in differentiating groups based on survival times, which is crucial in high-dimensional data where we aim to find a strong separation based on key features. Its simplicity and efficiency make it a practical choice when the primary goal is to maximize the separation of survival risks. The log-rank score split rule, a variation on the traditional log-rank rule, utilizes a scoring mechanism that assigns weights to each feature based on its survival difference contribution^48^. This rule is particularly useful when dealing with high-dimensional data, as it can identify nuanced effects and interactions among variables that may not be as apparent with the traditional log-rank rule. By focusing on weighted scores, this rule allows the model to capture subtle but potentially impactful variable interactions^72^. This can improve performance in complex datasets with overlapping or weak signal features, as it emphasizes the marginal contributions of features. The log-rank score rule was selected to complement the log-rank rule, as it enables the model to handle intricate, high-dimensional datasets more effectively by emphasizing subtle differences. This is particularly important in our analysis, where cytokine interactions may have non-obvious effects on survival outcomes.

SHAP values offer a comprehensive understanding of how individual cytokines contribute to HIV incidence prediction. By providing interpretable explanations for model predictions^73^, SHAP values enable researchers to identify cytokines with the most significant impact on HIV incidence. SHAP values offer several advantages in interpreting machine learning models. First, they are model agnostic, meaning they can be utilized to interpret any machine learning model, regardless of its architecture or complexity^55^. Additionally, SHAP values exhibit additivity, enabling the computation of the contribution of each feature to the final prediction independently and subsequently summing them. This additivity property facilitates efficient computation, even for high-dimensional datasets^74^. Moreover, SHAP values offer local accuracy, accurately reflecting the difference between the output of the expected model and the actual output for a specific input^75^. This provides a precise and localized interpretation of the model’s prediction for a given input. Furthermore, SHAP values demonstrate robustness to missing or irrelevant features for a prediction, as they are zero in such cases. This characteristic ensures that SHAP values remain reliable and unaffected by missing data while preventing irrelevant features from distorting the interpretation^74^. Lastly, SHAP values exhibit consistency, as they do not change when the model changes unless the contribution of a feature changes^76^. This consistency ensures that SHAP values provide a stable interpretation of the model’s behavior, even amidst changes in model architecture or parameters. Overall, SHAP values offer a consistent and objective means to gain insights into how the RSF models formulate predictions and which features exert the most substantial influence.

In the primary analysis of the dataset conducted by Karim et al.^16^ and Mansoor et al.^17^ the traditional Cox PH model was employed. This approach adjusted for potentially significant baseline covariates and did not use any cytokine profile in order to address the high dimensionality and complexity of the dataset. Other studies that analyzed the same data set^18–20^, selected fewer cytokines as their covariates thereby confounding the effect of other significant cytokine covariates. Our study has provided an alternative approach for analyzing high dimensional HIV survival data, particularly in the context of time-varying cytokine profiles. Our analysis revealed several cytokine profiles as strong positive predictors of HIV incidence, including tumor necrosis factor-alpha (TNF-A)^64^, basic fibroblast growth factor (BASIC-FGF)^77^, Interleukin (IL-5)^78^, monocyte chemotactic protein-3 (MCP-3)^79^ and EOTAXIN^80^. Additionally, baseline variables such as the frequency of condom use, treatment, number of partners, and number of times one had sex were identified as influential predictors^81–85^. Conversely, cytokines such as Interleukin (IL-1A, IL-1RA, IL-2, IL-2RA, IL-3, IL-6, IL-8, IL-12P40, IL-13, IL-15, IL-16), monokine induced interferon-gamma (MIG),^86^ granulocyte colony-stimulating factor (G-CSF), granulocyte-macrophage colony-stimulating factor (GM-CSF),^87^ Interferon-gamma (IFN-G), interferon alpha-2 (IFN-A2),^88^ induced protein 10 (IP-10)^89^, monocyte chemotactic protein-1 (MCP-1)^79^, macrophage inflammatory protein-1 (MIP-1B), RANTES,^90^ platelet-derived growth factor-BB (PDGF-BB)^91^, growth related oncogene-alpha (GRO-A)^92^, vascular endothelial growth factor (VEGF)^93^, hepatocyte growth factor (HGF)^94^, macrophage migration inhibitory factor (MIF)^95^, Stem cell factor (SCF)^94^, stromal cell-derived factor-1 (SDF-1A)^96^ and tumor necrosis factor (TNF)-related apoptosis-inducing ligand (TRAIL)^97^ were identified as strong negative predictors of HIV infection.

The concordance index and integrated Brier scores indicated excellent predictive accuracy of the RSF models, which was further supported by the interpretation of the SHAP values. There was consistency between the variable importance rankings and SHAP values, as the majority of the variables identified among the top 20 in variable importance were also ranked within the top 20 by SHAP values. While the SSVM analysis provided only the C-index, the RSF analysis offered a more comprehensive evaluation, including the calculation of variable importance, SHAP values, and various curves. Additionally, there are currently no available functions in R to perform such a comprehensive evaluation with SSVM. This broader analytical capability of RSF highlights its advantage over SSVM. The utilization of RSF models for analyzing cytokine profiles as predictors of HIV incidence has significant implications for HIV research and public health. By elucidating the immunological mechanisms underlying infection susceptibility, this study contributes to the development of novel biomarkers for HIV risk assessment which in turn may inform HIV acquisition prevention strategies such as vaccine development. Furthermore, the accurate prediction of HIV incidence using RSF models can inform the design and implementation of targeted and personalized interventions, ultimately reducing the burden of HIV/AIDS and improving public health outcomes^98^.

Both the SSVM and RSF models have their strengths in handling survival data, but they also come with specific limitations, particularly regarding overfitting, data requirements, computational cost, and generalizability. In the context of SVMs, overfitting can result from both an inadequate sample size and the complexity of the kernel function chosen^99^. The choice of kernel plays a pivotal role in the SVM’s performance. Given the infinite possibilities for kernel functions, selecting the appropriate kernel to match the underlying data distribution can be non-trivial^100^. A kernel that is too flexible may fit the noise of the training data, thus leading to poor performance on unseen data. The hybrid model approach in SSVM, while powerful, can overfit when the model tries to balance classification and regression tasks. Tuning of parameters like *gamma*.*mu* are essential but may not fully mitigate overfitting, particularly in small datasets^101^. Although RSF is inherently more resistant to overfitting due to ensemble averaging, it can still overfit if a large number of trees are grown without sufficient regularization. Furthermore, RSF’s reliance on bootstrap sampling could lead to overfitting on smaller datasets, as the same data points may repeatedly influence tree structure, reinforcing noise rather than signal^102^. Ensuring sufficient sample size and using tuning strategies like adjusting the *nodesize* and *ntree* parameters can help mitigate this.

The generalizability of the SSVM model is strongly affected by sample size. Because Survival SVM models aim to maximize the margin between classes, small samples can hinder the model’s ability to identify a clear margin. This sensitivity to sample size limits its generalizability, particularly when the training data does not adequately represent the broader population^103^. In contrast, RSF models, with their non-parametric structure, show some resilience to sample size constraints. However, they, too, struggle with generalizability in small or unbalanced datasets. For instance, when survival times are highly censored or event rates are low, RSF may not effectively capture the relationship between covariates and survival outcomes, as it relies on splitting rules that work best with well-represented events. Furthermore, RSF’s use of bootstrapped samples can cause underrepresented patterns in smaller datasets to be overlooked, leading to a model that may generalize poorly^104^. The computational cost of SSVM can be prohibitive, especially for large datasets or high-dimensional feature spaces. Kernel-based methods increase computational requirements, making SSVM challenging to scale for larger datasets or when real-time predictions are needed. While RSF is generally less computationally intensive than SSVM, its complexity grows with an increase in the number of trees (*ntree*) and the size of each tree. The choice of hyperparameters, such as *nsplit* and *nodesize*, also impacts computation. For example, a large nsplit can make each split more computationally demanding, especially if many trees are grown. However, RSF remains computationally manageable compared to SSVM for most high-dimensional datasets.

Applying SSVM and RSF models to high-dimensional data posed several computational challenges, including increased processing time, memory demands, and parameter optimization complexities. For the SSVM models, complex kernels like additive or radial basis function (RBF) are essential to transform and fit high-dimensional data but require substantial computational time. To manage this, the additive kernel and a hybrid approach were selected to balance model complexity with computational efficiency, reducing processing time compared to other kernel options. In the RSF model, the ensemble nature of the forest, building hundreds of trees with high-dimensional inputs, extended computation time, especially when the number of random splits and trees are high. To counter this, the *block*.*size* parameter was reduced to 1, ensuring each tree was computed individually, thereby managing memory use and limiting the system’s peak computational load. Using a smaller *nsplit* value (set to 5) and fewer trees ($ntree = 500$) allowed us to improve efficiency while maintaining model performance. Additionally, training processes were parallelized using all available cores (*ncores* set to $detectCores() - 1$), significantly accelerating model training. SSVM’s reliance on kernel matrices, which grow quadratically with sample size, created significant memory demands, especially with larger datasets. To manage this, we minimized kernel complexity by tuning the *gamma*.*mu* parameter. For the RSF model, memory usage also increased as each tree stored information on splits and nodes in high-dimensional data. To address this, we optimized the *nodesize* parameter to limit tree depth, thus reducing memory strain. Additionally, we ran code in smaller chunks and ensured no other programs were running in the background, maximizing available memory for processing.

The ablation study was a critical component of this research, as it allowed us to systematically evaluate the impact of grouped features on the predictive performance of our survival models. Given the high-dimensional, time-varying nature of the cytokine profiles, it was essential to determine an optimal set of features that could provide strong predictive power without unnecessarily increasing model complexity. This was particularly important because including too many features can lead to overfitting and increased computational demands. Overall, the ablation study demonstrated that using a well-selected subset of cytokine features, rather than the full set, could achieve comparable or even superior performance, emphasizing the importance of feature selection in machine learning models for biomedical research.

## Conclusion

In conclusion, this study demonstrated the effectiveness of applying machine learning survival models, specifically SSVM and RSF, to assess HIV risk using high-dimensional cytokine profiles and baseline factors. The RSF models consistently outperformed the SSVM models, especially when using the difference covariate model with the log-rank split rule, highlighting RSF’s capacity to handle complex, time-varying data while achieving high predictive accuracy. Our findings contribute to a deeper understanding of the role of cytokine profiles in HIV infection dynamics and highlight the importance of incorporating them into predictive models for HIV risk assessment. Moreover, the interpretability of RSF models, facilitated by measures such as VIMP and SHAP values, provides valuable insights into which cytokines increase or decrease HIV infection. Through our ablation study, we demonstrated that using a well-selected subset of cytokine features rather than the full set could achieve comparable or even superior performance. Overall, our study contributes to the growing body of literature on cytokine-based predictors of HIV incidence and underscores the utility of RSF models in survival analysis.

Further exploration of cytokine profiles may entail employing other ensemble methods for survival analysis, such as conditional inference forests and relative risk forests. Methods that enable the examination of time-varying covariates in their original, unaltered form are a potential area of further extension to improve the difference between the last observed and first measured cytokine values models. The analyses done in this paper may be extended and applied to other infectious diseases.

## Supplementary Information

**Supplementary information** The R code file that produced the results of the analysis is available in PDF. (pdf 291KB)

## Data Availability

Researchers wanting to access data from the completed CAPRISA studies are requested to complete a data request form. The form can be accessed at CAPRISA Studies.

## References

[1] Dembic, Z. *The cytokines of the immune system: the role of cytokines in disease related to immune response* (Academic Press, 2015).

[2] Ye, Q., Shao, W.-X., Xu, X.-J. & Yang, Y.-Z. The clinical application value of cytokines in treating infectious diseases. *PLoS ONE* **9**, e98745 (2014). 24887408 10.1371/journal.pone.0098745 PMC4041886

[3] Lin, D. Y. & Wei, L.-J. The robust inference for the cox proportional hazards model. *J. Am. Stat. Assoc.* **84**, 1074–1078 (1989).

[4] Baralou, V., Kalpourtzi, N. & Touloumi, G. Individual risk prediction: Comparing random forests with cox proportional-hazards model by a simulation study. *Biomet. J.* **65**, 2100380 (2023). 10.1002/bimj.202100380 36169048

[5] Rajula, H. S. R., Verlato, G., Manchia, M., Antonucci, N. & Fanos, V. Comparison of conventional statistical methods with machine learning in medicine: diagnosis, drug development, and treatment. *Medicina* **56**, 455 (2020). 32911665 10.3390/medicina56090455 PMC7560135

[6] Wang, P., Li, Y. & Reddy, C. K. Machine learning for survival analysis: A survey. *ACM Comput. Surv.(CSUR)* **51**, 1–36 (2019).

[7] Omurlu, I. K., Ture, M. & Tokatli, F. The comparisons of random survival forests and cox regression analysis with simulation and an application related to breast cancer. *Expert Syst. Appl.* **36**, 8582–8588 (2009).

[8] Weathers, B. & Cutler, R. D. *Comparision of survival curves between cox proportional hazards, random forests, and conditional inference forests in survival analysis* (Utah State University, Logan, UH, 2017).

[9] Datema, F. R. et al. Novel head and neck cancer survival analysis approach: random survival forests versus cox proportional hazards regression. *Head & Neck* **34**, 50–58 (2012). 21322080 10.1002/hed.21698

[10] Qiu, X. et al. A comparison study of machine learning (random survival forest) and classic statistic (cox proportional hazards) for predicting progression in high-grade glioma after proton and carbon ion radiotherapy. *Front. Oncology* **10**, 551420 (2020). 10.3389/fonc.2020.551420 PMC7662123 33194609

[11] Widodo, A. & Yang, B.-S. Machine health prognostics using survival probability and support vector machine. *Expert Syst. Appl.* **38**, 8430–8437 (2011).

[12] Pölsterl, S., Navab, N. & Katouzian, A. An efficient training algorithm for kernel survival support vector machines. *arXiv preprint*[SPACE]arXiv:1611.07054 (2016).

[13] Kiaee, F., Sheikhzadeh, H. & Mahabadi, S. E. Relevance vector machine for survival analysis. *IEEE Trans. Neural Netw. Learn. Syst.* **27**, 648–660 (2015). 25910258 10.1109/TNNLS.2015.2420611

[14] Lee, Y.-J. et al. Breast cancer survival and chemotherapy: A support vector machine analysis. *Discrete Math. Prob. Med. Appl.* **55**, 1–20 (1999).

[15] Pölsterl, S., Navab, N. & Katouzian, A. Fast training of support vector machines for survival analysis. In *Machine Learning and Knowledge Discovery in Databases: European Conference, ECML PKDD 2015, Porto, Portugal, September 7-11, 2015, Proceedings, Part II 15*, 243–259 (Springer, 2015).

[16] Abdool Karim, Q. et al. Effectiveness and safety of tenofovir gel, an antiretroviral microbicide, for the prevention of HIV infection in women. *Science* **329**, 1168–1174 (2010). 20643915 10.1126/science.1193748 PMC3001187

[17] Mansoor, L. E. et al. Adherence in the Caprisa 004 tenofovir gel microbicide trial. *AIDS Behav.* **18**, 811–819 (2014). 24643315 10.1007/s10461-014-0751-x PMC4017080

[18] Masson, L. et al. Genital inflammation and the risk of HIV acquisition in women. *Clin. Infect. Dis.* **61**, 260–269 (2015). 25900168 10.1093/cid/civ298 PMC4565995

[19] Naranbhai, V. et al. Innate immune activation enhances HIV acquisition in women, diminishing the effectiveness of tenofovir microbicide gel. *J. Infect. Dis.* **206**, 993–1001 (2012). 22829639 10.1093/infdis/jis465 PMC3501691

[20] Ignacio, R. A. B. *et al.* Dynamic immune markers predict hiv acquisition and augment associations with sociobehavioral factors for hiv exposure. *Iscience* **25** (2022). 10.1016/j.isci.2022.105632 PMC9722478 36483014

[21] Pickett, K. L., Suresh, K., Campbell, K. R., Davis, S. & Juarez-Colunga, E. Random survival forests for dynamic predictions of a time-to-event outcome using a longitudinal biomarker. *BMC Med. Res. Methodol.* **21**, 1–14 (2021). 34657597 10.1186/s12874-021-01375-x PMC8520610

[22] Ishwaran, H., Lauer, M. S., Blackstone, E. H., Lu, M. & Kogalur, U. B. Randomforestsrc: Random survival forests vignette (2021).

[23] Krzyziński, M., Spytek, M., Baniecki, H. & Biecek, P. Survshap (t): time-dependent explanations of machine learning survival models. *Knowl. Based Syst.* **262**, 110234 (2023). 10.1093/bioinformatics/btad723 PMC11025379 38039146

[24] Moncada-Torres, A., van Maaren, M. C., Hendriks, M. P., Siesling, S. & Geleijnse, G. Explainable machine learning can outperform cox regression predictions and provide insights in breast cancer survival. *Sci. Rep.* **11**, 6968 (2021). 33772109 10.1038/s41598-021-86327-7 PMC7998037

[25] Nohara, Y., Matsumoto, K., Soejima, H. & Nakashima, N. Explanation of machine learning models using Shapley additive explanation and application for real data in hospital. *Comput. Methods Prog. Biomed.* **214**, 106584 (2022). 10.1016/j.cmpb.2021.106584 34942412

[26] Liu, Z. *et al.* Efficient support vector machine method for survival prediction with seer data. In *Advances in Computational Biology*, 11–18 (Springer, 2010). 10.1007/978-1-4419-5913-3_2 20865481

[27] The r project for statistical computing (2024). Accessed: 2024-08-06.

[28] Vapnik, V. *The nature of statistical learning theory* (Springer science & business media, 2013).

[29] Karatzoglou, A., Smola, A., Hornik, K. & Zeileis, A. kernlab-an s4 package for kernel methods in r. *J. Stat. Softw.* **11**, 1–20 (2004).

[30] Scholkopf, B. & Smola, A. J. *Learning with kernels: support vector machines, regularization, optimization, and beyond* (MIT press, 2018).

[31] Evers, L. & Messow, C.-M. Sparse kernel methods for high-dimensional survival data. *Bioinformatics* **24**, 1632–1638 (2008). 18515276 10.1093/bioinformatics/btn253

[32] Cristianini, N. & Scholkopf, B. Support vector machines and kernel methods: the new generation of learning machines. *AI Mag.* **23**, 31–31 (2002).

[33] Liu, L., Shen, B. & Wang, X. Research on kernel function of support vector machine. In *Advanced Technologies, Embedded and Multimedia for Human-centric Computing: HumanCom and EMC 2013*, 827–834 (Springer, 2014).

[34] Shivaswamy, P. K., Chu, W. & Jansche, M. A support vector approach to censored targets. In: *Seventh IEEE International Conference on Data Mining (ICDM 2007)*, 655–660 (IEEE, 2007).

[35] Vapnik, V. N., Vapnik, V. *et al.**Statistical Learning Theory* (wiley New York, 1998).

[36] Van Belle, V., Pelckmans, K., Suykens, J. A. & Van Huffel, S. Support vector machines for survival analysis. In: *Proceedings of the Third International Conference on Computational Intelligence in Medicine and Healthcare (cimed2007)*, 1–8 (2007).

[37] Van Belle, V., Pelckmans, K., Suykens, J. A. & Van Huffel, S. Survival svm: a practical scalable algorithm. In *ESANN*, 89–94 (2008).

[38] Van Belle, V., Pelckmans, K., Van Huffel, S. & Suykens, J. A. Support vector methods for survival analysis: a comparison between ranking and regression approaches. *Artif. Intell. Med.* **53**, 107–118 (2011). 21821401 10.1016/j.artmed.2011.06.006

[39] Fouodo, C. J., König, I. R., Weihs, C., Ziegler, A. & Wright, M. N. Support vector machines for survival analysis with R. *R J.* **10**, 412–423 (2018).

[40] Ishwaran, H., Kogalur, U. B., Blackstone, E. H. & Lauer, M. S. Random survival forests. *Annals Appl. Stat.* **2**, 840–860 (2008).

[41] Breiman, L. Random forests. *Mach. Learn.* **45**, 5–32 (2001).

[42] Wang, H. & Li, G. A selective review on random survival forests for high dimensional data. *Quant. Bio-Sci.* **36**, 85 (2017). 10.22283/qbs.2017.36.2.85 PMC6364686 30740388

[43] Ishwaran, H., Kogalur, U. B., Chen, X. & Minn, A. J. Random survival forests for high-dimensional data. *Stat. Anal. Data Mining: ASA Data Sci. J.* **4**, 115–132 (2011).

[44] Jiang, S. Prediction based on random survival forest. *Am. J. Biomed. Sci. Res.* **6**, 109–111 (2019).

[45] Ishwaran, H., Kogalur, U. B. & Kogalur, M. U. B. Package â€˜randomforestsrcâ€™. *Breast* **6** (2022).

[46] Nelson, W. Theory and applications of hazard plotting for censored failure data. *Technometrics* **14**, 945–966 (1972).

[47] Ehrlinger, J. ggrandomforests: Exploring random forest survival. *arXiv preprint*[SPACE]arXiv:1612.08974 (2016).

[48] Hothorn, T. & Lausen, B. On the exact distribution of maximally selected rank statistics. *Comput. Stat. Data Anal.* **43**, 121–137 (2003).

[49] Mohammed, M., Mboya, I. B., Mwambi, H., Elbashir, M. K. & Omolo, B. Predictors of colorectal cancer survival using cox regression and random survival forests models based on gene expression data. *PLoS ONE* **16**, e0261625 (2021). 34965262 10.1371/journal.pone.0261625 PMC8716055

[50] Harrell, F. E., Califf, R. M., Pryor, D. B., Lee, K. L. & Rosati, R. A. Evaluating the yield of medical tests. *JAMA* **247**, 2543–2546 (1982). 7069920

[51] May, M. et al. Development and validation of a prognostic model for survival time data: application to prognosis of HIV positive patients treated with antiretroviral therapy. *Stat. Med.* **23**, 2375–2398 (2004). 15273954 10.1002/sim.1825

[52] Kattan, M. W., Hess, K. R. & Beck, J. R. Experiments to determine whether recursive partitioning (cart) or an artificial neural network overcomes theoretical limitations of cox proportional hazards regression. *Comput. Biomed. Res.* **31**, 363–373 (1998). 9790741 10.1006/cbmr.1998.1488

[53] Graf, E., Schmoor, C., Sauerbrei, W. & Schumacher, M. Assessment and comparison of prognostic classification schemes for survival data. *Stat. Med.* **18**, 2529–2545 (1999). 10474158 10.1002/(sici)1097-0258(19990915/30)18:17/18<2529::aid-sim274>3.0.co;2-5

[54] Hallett, M., Fan, J., Su, X., Levine, R. & Nunn, M. E. Random forest and variable importance rankings for correlated survival data, with applications to tooth loss. *Stat. Model.* **14**, 523–547 (2014).

[55] Lundberg, S. M. & Lee, S.-I. A unified approach to interpreting model predictions. *Advances in neural information processing systems* **30** (2017).

[56] Redelmeier, A., Jullum, M. & Aas, K. Explaining predictive models with mixed features using shapley values and conditional inference trees. In *Machine Learning and Knowledge Extraction: 4th IFIP TC 5, TC 12, WG 8.4, WG 8.9, WG 12.9 International Cross-Domain Conference, CD-MAKE 2020, Dublin, Ireland, August 25–28, 2020, Proceedings 4*, 117–137 (Springer, 2020).

[57] Chalkiadakis, G., Elkind, E. & Wooldridge, M. *Computational aspects of cooperative game theory* (Springer Nature, 2022).

[58] Elkind, E. & Rothe, J. Cooperative game theory. *Economics and computation: An introduction to algorithmic game theory, computational social choice, and fair division* 34, 1-39.(2016).

[59] Wang, Y. et al. Cell graph neural networks enable the precise prediction of patient survival in gastric cancer. *NPJ Precis. Oncol.* **6**, 45 (2022). 35739342 10.1038/s41698-022-00285-5 PMC9226174

[60] Taylor, J. M. Random survival forests. *J. Thorac. Oncol.* **6**, 1974–1975 (2011). 22088987 10.1097/JTO.0b013e318233d835

[61] Spytek, M. et al. Survex: An r package for explaining machine learning survival models. *Bioinformatics* **39**, btad723 (2023). 38039146 10.1093/bioinformatics/btad723 PMC11025379

[62] Mogensen, U. B., Ishwaran, H. & Gerds, T. A. Evaluating random forests for survival analysis using prediction error curves. *J. Stat. Softw.* **50**, 1 (2012). 25317082 10.18637/jss.v050.i11 PMC4194196

[63] Meager, A. & Wadhwa, M. An overview of cytokine regulation of inflammation and immunity. *eLS* (2013).

[64] Reuter, M. A., Pombo, C. & Betts, M. R. Cytokine production and dysregulation in HIV pathogenesis: Lessons for development of therapeutics and vaccines. *Cytokine Growth Factor Rev.* **23**, 181–191 (2012). 22743036 10.1016/j.cytogfr.2012.05.005 PMC3582023

[65] Roberts, L. et al. Genital tract inflammation during early HIV-1 infection predicts higher plasma viral load set point in women. *J. Infect. Dis.* **205**, 194–203 (2012). 22190580 10.1093/infdis/jir715 PMC3244362

[66] Breen, E. C. Pro-and anti-inflammatory cytokines in human immunodeficiency virus infection and acquired immunodeficiency syndrome. *Pharmacol. Therap.* **95**, 295–304 (2002). 12243799 10.1016/s0163-7258(02)00263-2

[67] Seder, R. A., Grabstein, K. H., Berzofsky, J. A. & McDyer, J. F. Cytokine interactions in human immunodeficiency virus-infected individuals: Roles of interleukin (il)-2, il-12, and il-15. *J. Exp. Med.* **182**, 1067–1077 (1995). 7561680 10.1084/jem.182.4.1067 PMC2192305

[68] Veugelers, P. J. et al. Models of survival in HIV infection and their use in the quantification of treatment benefits. *Am. J. Epidemiol.* **148**, 487–496 (1998). 9737561 10.1093/oxfordjournals.aje.a009674

[69] Hamid, O., Tapak, M., Poorolajal, J., Amini, P. & Tapak, L. Application of random survival forest for competing risks in prediction of cumulative incidence function for progression to aids. *Epidemiology, Biostatistics, and Public Health* **14** (2017).

[70] Pozo Rodríguez, J. d. Use of machine learning algorithms for analysing viral cure after antiretroviral treatment in HIV+ patients (2021).

[71] Wei, F. et al. Machine learning for prediction of immunotherapeutic outcome in non-small-cell lung cancer based on circulating cytokine signatures. *J. Immunotherap. Cancer* **11**, e006788 (2023). 10.1136/jitc-2023-006788 PMC10347453 37433717

[72] Ishwaran, H. The effect of splitting on random forests. *Mach. Learn.* **99**, 75–118 (2015). 28919667 10.1007/s10994-014-5451-2 PMC5599182

[73] Aas, K., Jullum, M. & Løland, A. Explaining individual predictions when features are dependent: More accurate approximations to shapley values. *Artif. Intell.* **298**, 103502 (2021).

[74] Awan, A.A. An introduction to shap values and machine learning interpretability (2023).

[75] Li, Z. Extracting spatial effects from machine learning model using local interpretation method: An example of Shap and Xgboost. *Comput. Environ. Urban Syst.* **96**, 101845 (2022).

[76] Mi, J.-X., Li, A.-D. & Zhou, L.-F. Review study of interpretation methods for future interpretable machine learning. *IEEE Access* **8**, 191969–191985 (2020).

[77] Ray, P. E., Liu, X.-H., Xu, L. & Rakusan, T. Basic fibroblast growth factor in HIV-associated hemolytic uremic syndrome. *Pediatr. Nephrol.* **13**, 586–593 (1999). 10460507 10.1007/s004670050749

[78] Shete, A. et al. High il-5 levels possibly contributing to HIV viremia in virologic non-responders at one year after initiation of anti-retroviral therapy. *Microb. Pathog.* **143**, 104117 (2020). 32135221 10.1016/j.micpath.2020.104117

[79] Modi, W. S. et al. Mcp-1-Mcp-3-Eotaxin gene cluster influences HIV-1 transmission. *AIDS* **17**, 2357–2365 (2003). 14571188 10.1097/00002030-200311070-00011

[80] Lamoury, F. M. et al. HIV infection is associated with higher levels of monocyte chemoattractant protein-1 and Eotaxin among people with recent hepatitis c virus infection. *BMC Infect. Dis.* **16**, 1–9 (2016). 27246604 10.1186/s12879-016-1567-2 PMC4888248

[81] Kassanjee, R. et al. HIV incidence estimation among female sex workers in South Africa: A multiple methods analysis of cross-sectional survey data. *The Lancet HIV* **9**, e781–e790 (2022). 36075252 10.1016/S2352-3018(22)00201-6 PMC9626386

[82] Anderegg, N., Slabbert, M., Buthelezi, K. & Johnson, L. F. Increasing age and duration of sex work among female sex workers in South Africa and implications for HIV incidence estimation: Bayesian evidence synthesis and simulation exercise. *Infect. Dis. Model.* **9**, 263–277 (2024). 38323073 10.1016/j.idm.2024.01.006 PMC10844672

[83] Wang, H. et al. HIV incidence and associated risk factors among female sex workers in a high HIV-prevalence area of China. *Sex. Transm. Dis.* **39**, 835–841 (2012). 23064531 10.1097/OLQ.0b013e318266b241

[84] Dunkle, K. L. et al. Transactional sex among women in Soweto, South Africa: prevalence, risk factors and association with HIV infection. *Social Sci. Med.* **59**, 1581–1592 (2004). 10.1016/j.socscimed.2004.02.003 15279917

[85] Bazzi, A. R. et al. Incidence and predictors of HIV and sexually transmitted infections among female sex workers and their intimate male partners in northern mexico: a longitudinal, multilevel study. *Am. J. Epidemiol.* **181**, 723–731 (2015). 25769307 10.1093/aje/kwu340 PMC4408950

[86] Kedzierska, K. & Crowe, S. M. Cytokines and HIV-1: Interactions and clinical implications. *Antiviral Chem. Chemotherap.* **12**, 133–150 (2001). 10.1177/095632020101200301 12959322

[87] Frumkin, L. R. Role of granulocyte colony-stimulating factor and granulocyte-macrophage colony-stimulating factor in the treatment of patients with hiv infection. *Curr. Opin. Hematol.* **4**, 200–206 (1997). 9209837 10.1097/00062752-199704030-00008

[88] Roff, S. R., Noon-Song, E. N. & Yamamoto, J. K. The significance of interferon- in HIV-1 pathogenesis, therapy, and prophylaxis. *Front. Immunol.* **4**, 498 (2014). 24454311 10.3389/fimmu.2013.00498 PMC3888948

[89] Lei, J., Yin, X., Shang, H. & Jiang, Y. Ip-10 is highly involved in HIV infection. *Cytokine* **115**, 97–103 (2019). 30472104 10.1016/j.cyto.2018.11.018

[90] Cocchi, F. et al. Identification of rantes, mip-1, and mip-1 as the major HIV-suppressive factors produced by cd8+ t cells. *Science* **270**, 1811–1815 (1995). 8525373 10.1126/science.270.5243.1811

[91] Bethel-Brown, C. et al. HIV-1 tat-mediated induction of platelet-derived growth factor in astrocytes: Role of early growth response gene 1. *J. Immunol.* **186**, 4119–4129 (2011). 21368226 10.4049/jimmunol.1002235 PMC3110059

[92] Lane, B. R. et al. Interleukin-8 stimulates human immunodeficiency virus type 1 replication and is a potential new target for antiretroviral therapy. *J. Virol.* **75**, 8195–8202 (2001). 11483765 10.1128/JVI.75.17.8195-8202.2001 PMC115064

[93] Bussolino, F., Mitola, S., Serini, G., Barillari, G. & Ensoli, B. Interactions between endothelial cells and HIV-1. *Int. J. Biochem. Cell Biol.* **33**, 371–390 (2001). 11312107 10.1016/s1357-2725(01)00024-3

[94] Bordoni, V. et al. Impact of art on dynamics of growth factors and cytokines in primary HIV infection. *Cytokine* **125**, 154839 (2020). 31542514 10.1016/j.cyto.2019.154839

[95] Delaloye, J. et al. Increased macrophage migration inhibitory factor (MIF) plasma levels in acute HI-1 infection. *Cytokine* **60**, 338–340 (2012). 22898393 10.1016/j.cyto.2012.07.027

[96] Ikegawa, M. et al. Elevated plasma stromal cell-derived factor 1 protein level in the progression of HIV type 1 infection/aids. *AIDS Res. Hum. Retroviruses* **17**, 587–595 (2001). 11375054 10.1089/088922201300119680

[97] Miura, Y. et al. Critical contribution of tumor necrosis factor-related apoptosis-inducing ligand (trail) to apoptosis of human cd4+ t cells in HIV-1-infected Hu-Pbl-nod-Scid mice. *J. Exp. Med.* **193**, 651–660 (2001). 11238596 10.1084/jem.193.5.651 PMC2193390

[98] Read, S. W., Kim, P., Marovich, M., Dieffenbach, C. W. & Fauci, A. S. Forty years of investment in HIV research: Progress towards ending the HIV pandemic and preparation for future pandemics. *African J. Reprod. Gynaecol. Endoscopy* **25**, e26039 (2022). 10.1002/jia2.26039 PMC9709723 36448551

[99] Cawley, G. C. & Talbot, N. L. On over-fitting in model selection and subsequent selection bias in performance evaluation. *J. Mach. Learn. Res.* **11**, 2079–2107 (2010).

[100] Karamizadeh, S., Abdullah, S. M., Halimi, M., Shayan, J. & javad Rajabi, M. Advantage and drawback of support vector machine functionality. In: *2014 International Conference on Computer, Communications, and Control Technology (I4CT)*, 63–65 (IEEE, 2014).

[101] Cawley, G. C. & Talbot, N. L. Preventing over-fitting during model selection via Bayesian regularisation of the hyper-parameters. *J. Mach. Learn. Res.* **8**, 841–861 (2007).

[102] Belkin, M., Hsu, D. J. & Mitra, P. Overfitting or perfect fitting? risk bounds for classification and regression rules that interpolate. *Adv. Neural Inf. Process. Syst.* **31** (2018).

[103] Rajput, D., Wang, W.-J. & Chen, C.-C. Evaluation of a decided sample size in machine learning applications. *BMC Bioinformatics* (2023). 10.1186/s12859-023-05156-9 PMC9926644 36788550

[104] Yosefian, I., Mosa Farkhani, E. & Baneshi, M. R. Application of random forest survival models to increase generalizability of decision trees: A case study in acute myocardial infarction. *Comput. Math. Methods Med.* **2015**, 576413 (2015). 26858773 10.1155/2015/576413 PMC4698527

