# Supplementary material for: Cytokine profiles as predictors of HIV incidence using machine learning survival models and statistical interpretable techniques
Source: Sci Rep. 2024 Dec 2;14:29895. doi: 10.1038/s41598-024-81510-y (PMC11612445; doi:10.1038/s41598-024-81510-y)
Supplement: Supplementary file 1 — Supplementary information The R code file that produced the results of the analysis is available in PDF. (pdf 291KB) [file 41598_2024_81510_MOESM1_ESM.pdf]

Machine learning: Survival Support Vector Machine and Random Survival Forest Analysis code

Sarah Ogutu

2024-11-06

Load required packages

```
suppressPackageStartupMessages({
  library(readxl)
  library(dplyr)
  library(tidyverse)
  library(survival)
  library(survivalsvm)
  library(doParallel)
  library(ggplot2)
  library(RColorBrewer)
  library(parallel)
  library(randomForestSRC)
  library(ggRandomForests)
  library(pec)
  library(party)
  library(rms)
  library(survex)
  library(ranger)
  library(ggfortify)
  library(survminer)
  library(ggsurvfit)
  library(naniar)
  library(Hmisc)
  library(plyr)
  library(caret)
  library(gridExtra)
  library(viridis)
  library(ROCR)
  library(grImport2)
  library(kernelshap)
  library(shapviz)
  library(ggbeeswarm)
  library(kableExtra)
  library(xtable)
  library(knitr)
})
```

Importing pre-processed datasets

```
# use code chunks from Data_Pre_processing.rmd file
# the chunks are labeled mean and diff respectively

########### Mean model dataset ##########
invisible(knitr::purl("Data_Pre_Processing.Rmd", output="mean", quiet=TRUE))
read_chunk("mean")

########### Difference model dataset ##########
invisible(knitr::purl("Data_Pre_Processing.Rmd", output="diff", quiet=TRUE))
read_chunk("diff")
```

Data partitioning: Train set and test set

```
# Mean Model
set.seed(32024)
trainIndex <- createDataPartition(Base_surv_analysis18.1$HIV,p=.80,
                                  list=FALSE)
trainData <- Base_surv_analysis18.1[trainIndex,]
testData  <- Base_surv_analysis18.1[-trainIndex,]

# Difference model
set.seed(32024)
trainIndex_d <- createDataPartition(Base_surv_analysis21.1$HIV,p=.80,
                                    list=FALSE)
trainData_d <- Base_surv_analysis21.1[trainIndex_d,]
testData_d  <- Base_surv_analysis21.1[-trainIndex,]

# setting multicores to run random forest
ncores <- detectCores() - 1
cl <- makeCluster(ncores)
registerDoParallel(cl)
```

Survival Support Vector Machine (SSVM)

Mean model

```
set.seed(32024)
survsvm.HIVtrain <- survivalsvm(Surv(months, HIV) ~ ., data = trainData,
                                type = "hybrid", gamma.mu = c(0.5,0.5),
                                diff.meth = "makediff1",
                                opt.meth = "quadprog",
                                kernel = "add_kernel")

predict_HIV <- predict(object = survsvm.HIVtrain, newdata = testData)
Hmisc::rcorr.cens(predict_HIV$predicted,
                  Surv(testData$months, testData$HIV))
```

Difference model

```r
set.seed(32024)
survsvm.HIVtrain1 <- survivalsvm(Surv(months, HIV) ~ ., data = trainData_d,
                                 type = "hybrid", gamma.mu = c(0.5,0.5),
                                 diff.meth = "makediff1",
                                 opt.meth = "quadprog",
                                 kernel = "add_kernel")

predict_HIV1 <- predict(object = survsvm.HIVtrain1, newdata = testData_d)
Hmisc::rcorr.cens(predict_HIV1$predicted,
                  Surv(testData_d$months, testData_d$HIV))
```

Random Survival Forest Models (RSF)

Mean Model

```r
# Log-rank
set.seed(32024)
rsf_logrank <- rfsrc(Surv(months, HIV)~., trainData,
             ntree = 500, nodesize = 50, nsplit = 5,
             splitrule = "logrank", block.size = 1, importance = TRUE)
print(rsf_logrank)

# Log-rank-score
set.seed(32024)
rsf_logrankscore <- rfsrc(Surv(months, HIV)~., trainData,
                     ntree = 500, nodesize = 50, nsplit = 5,
                     splitrule = "logrankscore", block.size = 1,
                     importance = TRUE)
print(rsf_logrankscore)
```

Difference Model

```r
# Log-rank
set.seed(32024)
rsf_logrank_d <- rfsrc(Surv(months, HIV)~., trainData_d,
             ntree = 500, nodesize = 50, nsplit = 5,
             splitrule = "logrank", block.size = 1, importance = TRUE)
print(rsf_logrank_d)

# Log-rank-score
set.seed(32024)
rsf_logrankscore_d <- rfsrc(Surv(months, HIV)~., trainData_d,
                     ntree = 500, nodesize = 50,
                     nsplit = 5, splitrule = "logrankscore",
                     block.size = 1, importance = TRUE)
print(rsf_logrankscore_d)
```

Random Survival Forest plots

OOB error rate plot

```r
########## Mean Model ##########
# log-rank
e1 <- gg_error(rsf_logrank)
plogrank <- ggplot(e1, aes(x = ntree, y = error)) +
  labs(x="Number of Trees") + labs(y="Error Rate (log-rank mean model)") +
  theme_minimal() +
  geom_line(linewidth=1) +
  theme(axis.text = element_text(size = 13),
        axis.title = element_text(size = 13))

# log-rank-score
e2 <- gg_error(rsf_logrankscore)
plogrank_score <- ggplot(e2, aes(x = ntree, y = error)) +
  labs(x="Number of Trees") +
  labs(y="Error Rate (log-rank-score mean model)") +
  theme_minimal() +
  geom_line(linewidth=1) +
  theme(axis.text = element_text(size = 13),
        axis.title = element_text(size = 13))

########## Difference Model ##########
# log-rank
e1_d <- gg_error(rsf_logrank_d)
plogrank_d <- ggplot(e1_d, aes(x = ntree, y = error)) +
  labs(x="Number of Trees") +
  labs(y="Error Rate (log-rank difference model)") +
  theme_minimal() +
  geom_line(linewidth=1) +
  theme(axis.text = element_text(size = 13),
        axis.title = element_text(size = 13))

# log-rank-score
e2_d <- gg_error(rsf_logrankscore_d)
plogrank_score_d <- ggplot(e2_d, aes(x = ntree, y = error)) +
  labs(x="Number of Trees") +
  labs(y="Error Rate (log-rank-score difference model)") +
  theme_minimal() +
  geom_line(linewidth=1) +
  theme(axis.text = element_text(size = 13),
        axis.title = element_text(size = 13))

########## Grid arrange as one plot ##########
OOB_error_plot <- grid.arrange(plogrank, plogrank_score,
                               plogrank_d, plogrank_score_d,
                               nrow =2, ncol = 2)
ggsave("Figure1.pdf", plot = OOB_error_plot, width = 9, height = 9,
       units = "in", dpi = 600, device = "pdf")
```

Variable importance plot

```r
# rename the variables
new_names <- c("Treat" = "Treatment",
               "p2v18_REG_PARTNER_LIVE_TOGETHER"="Live with partner",
               "p2v22_HIGHEST_EDUCATION"="Highest Education",
               "p3v8_SELF_GEN_INCOME"="Self Gen Income",
               "p3v9_SALARY"="Salary", "p3v10_HUSBAND"="Husband's Income",
               "p3v11_SOCIAL_GRANTS"="Social Grants",
               "p3v13_OTHER_INCOME_SOURCE"="Other Income",
               "p3v16_AMOUNT_INCOME"="Income Amount",
               "p5v9_LENGTH_IN_DBNVUL"="Time in Durban (years)",
               "age1"="Age at enrollment", "marital"="Marital Status",
               "agedebu"="Age at Debut",
               "p17v10_PARTNERS"="Total Partners",
               "p17v11_YEAR_STABLE"="Stable Partners (past year)",
               "p17v12_YEAR_CASUAL"="Casual Partners (past year)",
               "p17v13_30DAYS_STABLE"="Stable Partners (30 Days)",
               "p17v14_30DAYS_CASUAL"="Casual Partners (30 Days)",
               "p17v15_SEX_30DAYS"="Times Sex (30 Days)",
               "p17v26_AGE_OLDEST_SEX_PART"="Oldest Partner",
               "p17v28_SEX_PART_HAVE_OTHER"="Sex Partner have other",
               "p18v15_FREQ_CONDOM_USE"="Condom Use",
               "p19v14_ABNORMAL_DISCHARGE"="Abnormal Discharge",
               "p18v8_SEX_PART_TEST_POS"="Sex Partner test positive")
########## Mean model ##########
# log-rank
varimp.rsflr <- gg_vimp(rsf_logrank)

varimp.rsflr <- varimp.rsflr %>%
  mutate(vars = str_replace_all(vars, new_names))

varimp.rsflr <- varimp.rsflr[order(-varimp.rsflr$vimp),]

p_varimp.rsflr <-  varimp.rsflr %>%
  arrange(vimp) %>%
  mutate(name = factor(vars, levels = vars)) %>%
  ggplot(aes(x = name, y = vimp)) +
  geom_segment(aes(xend = name, yend = 0), size = 2, color = "black") +
  geom_point(size = 4, color = "orange") +
  coord_flip() +
  theme_bw() +
  labs(tag = "a", x = "", y = "Permutation Importance") +
  theme(axis.text = element_text(size = 8),
        axis.title = element_text(size = 10),
        axis.ticks = element_line(linewidth = 2))
# log-rank score
varimp.rsflrs <- gg_vimp(rsf_logrankscore)

varimp.rsflrs <- varimp.rsflrs %>%
  mutate(vars = str_replace_all(vars, new_names))

varimp.rsflrs <- varimp.rsflrs[order(-varimp.rsflrs$vimp),]
```

```
p_varimp.rsflrs <- varimp.rsflrs %>%
  arrange(vimp) %>%
  mutate(name = factor(vars, levels = vars)) %>%
  ggplot(aes(x = name, y = vimp)) +
  geom_segment(aes(xend = name, yend = 0), size = 2, color = "black") +
  geom_point(size = 4, color = "orange") +
  coord_flip() +
  theme_bw() +
  labs(tag = "b", x = "", y = "Permutation Importance") +
  theme(axis.text = element_text(size = 8),
        axis.title = element_text(size = 10),
        axis.ticks = element_line(linewidth = 2))

varimp_both_lr_lrs <- grid.arrange(p_varimp.rsflr, p_varimp.rsflrs,
                                   nrow =1 , ncol = 2)

ggsave("Figure 2.pdf", plot = varimp_both_lr_lrs,
       width = 9, height = 12, units = "in", dpi = 600, device = "pdf")

##########  Difference model ##########
# log-rank
varimp.rsflr_d <- gg_vimp(rsf_logrank_d)

varimp.rsflr_d <- varimp.rsflr_d %>%
  mutate(vars = str_replace_all(vars, new_names))

varimp.rsflr_d <- varimp.rsflr_d[order(-varimp.rsflr_d$vimp),]

p_varimp.rsflr_d <-  varimp.rsflr_d %>%
  arrange(vimp) %>%
  mutate(name = factor(vars, levels = vars)) %>%
  ggplot(aes(x = name, y = vimp)) +
  geom_segment(aes(xend = name, yend = 0), size = 2, color = "black") +
  geom_point(size = 4, color = "orange") +
  coord_flip() +
  theme_bw() +
  labs(tag = "a", x = "", y = "Permutation Importance") +
  theme(axis.text = element_text(size = 8),
        axis.title = element_text(size = 10),
        axis.ticks = element_line(linewidth = 2))

# log-rank score

varimp.rsflrs_d <- gg_vimp(rsf_logrankscore_d)

varimp.rsflrs_d <- varimp.rsflrs_d %>%
  mutate(vars = str_replace_all(vars, new_names))

varimp.rsflrs_d <- varimp.rsflrs_d[order(-varimp.rsflrs_d$vimp),]

p_varimp.rsflrs_d <- varimp.rsflrs_d %>%
  arrange(vimp) %>%
  mutate(name = factor(vars, levels = vars)) %>%
```

```
  ggplot(aes(x = name, y = vimp)) +
  geom_segment(aes(xend = name, yend = 0), size = 2, color = "black") +
  geom_point(size = 4, color = "orange") +
  coord_flip() +
  theme_bw() +
  labs(tag = "b", x = "", y = "Permutation Importance") +
  theme(axis.text = element_text(size = 8),
        axis.title = element_text(size = 10),
        axis.ticks = element_line(linewidth = 2))
varimp_both_lr_lrs_d <- grid.arrange(p_varimp.rsflr_d, p_varimp.rsflrs_d,
                                     nrow=1, ncol=2)

ggsave("Figure3.pdf", plot = varimp_both_lr_lrs_d,
       width = 9, height = 12, units = "in", dpi = 600, device = "pdf")
```

Explainable Machine learning for Random Survival Forest model

```
# creating an explainer
########## Mean Model ##########
# log-rank
set.seed(32024)
rsf_explainer <- survex::explain(
  rsf_logrank, data = trainData[, -which(
    names(trainData) %in% c("months","HIV"))], y =
    Surv(trainData$months, trainData$HIV), label = "RSF-logrank")
# log-rank-score
set.seed(32024)
rsfscore_explainer <- survex::explain(
  rsf_logrankscore, data = trainData[, -which(
    names(trainData) %in% c("months","HIV"))], y =
    Surv(trainData$months, trainData$HIV), label = "RSF-logrankscore")

########## Difference Model ##########
# log-rank
set.seed(32024)
rsf_explainer_d <- survex::explain(
  rsf_logrank_d, data = trainData_d[, -which(
    names(trainData_d) %in% c("months","HIV"))], y =
    Surv(trainData_d$months, trainData_d$HIV), label = "RSF-logrank_d")
# log-rank-score
set.seed(32024)
rsfscore_explainer_d <- survex::explain(
  rsf_logrankscore_d, data = trainData_d[, -which(
    names(trainData_d) %in% c("months","HIV"))], y =
    Surv(trainData_d$months, trainData_d$HIV), label = "RSF-logrankscore_d")
```

AUC and Brier Scores: Model performance

```
########## Mean Model #########
# log-rank
```

```r
set.seed(32024)
rsf_perform <- model_performance(rsf_explainer)
plot(rsf_perform)
rsf_perform_df <- data.frame(time = rsf_perform$eval_times,
                             BS = rsf_perform$result$`Brier score`,
                             AUC = rsf_perform$result$`C/D AUC`,
                             label = "RSF")

# log-rank-score
set.seed(32024)
rsfs_perform <- model_performance(rsfscore_explainer)
plot(rsfs_perform)
rsfs_perform_df <- data.frame(time = rsfs_perform$eval_times,
                              BS = rsfs_perform$result$`Brier score`,
                              AUC = rsfs_perform$result$`C/D AUC`,
                              label = "RSFS")
## Merge the data frames
model_perform_df1 <- rbind(rsf_perform_df, rsfs_perform_df)
model_perform_df1$label <- as.factor(model_perform_df1$label)
levels(model_perform_df1$label) <- c("RSF-logrank_M", "RSF-logrankscore_M")
## reshape to long format
model_perform_df_long1 <- model_perform_df1 %>% pivot_longer(cols=c('BS','AUC'),
                                  names_to='Metric',
                                  values_to='Value')
model_perform_df_long1 <- model_perform_df_long1 %>% arrange(label, Metric)
model_perform_df_long1$Metric <- as.factor(model_perform_df_long1$Metric)
levels(model_perform_df_long1$Metric) <- c("AUC", "Brier Score")

## plot the performance metrics over time
## AUC - Brier Score
AUC_BS_plot1 <- model_perform_df_long1 %>%
  ggplot(aes(color=label)) +
  geom_line(mapping=aes(x=time, y=Value), linewidth = 2) +
  scale_color_viridis(discrete = TRUE) +
  theme_bw() +
  labs(x = "Survival Time (Months)", y = "Metric value") +
  theme(axis.text = element_text(size = 14),
        axis.title = element_text(size = 15),
        legend.position = "top", legend.title = element_text(size=14),
        legend.direction = "horizontal",
        legend.text = element_text(size = 16),
        strip.text.x = element_text(size = 15),
        strip.text.y = element_text(size = 15),
        panel.grid.major = element_blank(),
        panel.grid.minor = element_blank()) +
  facet_grid(Metric~., scales = "free") +
  guides(color = guide_legend(title = ""), fill = "none")
```

```r
########## Difference Model ##########
# log-rank
set.seed(32024)
rsf_perform_d <- model_performance(rsf_explainer_d)
plot(rsf_perform_d)
```

```r
rsf_perform_df_d <- data.frame(time = rsf_perform_d$eval_times,
                               BS = rsf_perform_d$result$`Brier score`,
                               AUC = rsf_perform_d$result$`C/D AUC`,
                               label = "RSF")

#log-rank-score
set.seed(32024)
rsfs_perform_d <- model_performance(rsfscore_explainer_d)
plot(rsfs_perform_d)
rsfs_perform_df_d <- data.frame(time = rsfs_perform_d$eval_times,
                                BS = rsfs_perform_d$result$`Brier score`,
                                AUC = rsfs_perform_d$result$`C/D AUC`,
                                label = "RSFS")

## merge the data frames
model_perform_df2 <- rbind(rsf_perform_df_d, rsfs_perform_df_d)
model_perform_df2$label <- as.factor(model_perform_df2$label)
levels(model_perform_df2$label) <- c("RSF-logrank_D", "RSF-logrankscore_D")
## reshape to long format
model_perform_df_long2 <- model_perform_df2 %>% pivot_longer(cols=c('BS','AUC'),
                                  names_to='Metric',
                                  values_to='Value')
model_perform_df_long2 <- model_perform_df_long2 %>% arrange(label, Metric)
model_perform_df_long2$Metric <- as.factor(model_perform_df_long2$Metric)
levels(model_perform_df_long2$Metric) <- c("AUC", "Brier Score")

## plot the performance metrics over time
## AUC - Brier Score
AUC_BS_plot2 <- model_perform_df_long2 %>%
  ggplot(aes(color=label)) +
  geom_line(mapping=aes(x=time, y=Value), linewidth = 2) +
  scale_color_viridis(discrete = TRUE) +
  theme_bw() +
  labs(x = "Survival Time (Months)", y = "Metric value") +
  theme(axis.text = element_text(size = 14),
        axis.title = element_text(size = 15),
        legend.position = "top", legend.title = element_text(size=14),
        legend.direction = "horizontal",
        legend.text = element_text(size = 16),
        strip.text.x = element_text(size = 15),
        strip.text.y = element_text(size = 15),
        panel.grid.major = element_blank(),
        panel.grid.minor = element_blank()) +
  facet_grid(Metric~., scales = "free") +
  guides(color = guide_legend(title = ""), fill = "none")
```

```r
### Combined plots
#plot(rsf_perform, rsfs_perform, rsf_perform_d, rsfs_perform_d)# default plot
AUC_BS_curve <- grid.arrange(AUC_BS_plot1, AUC_BS_plot2, nrow = 1, ncol = 2)
ggsave("Figure4.pdf", plot = AUC_BS_curve, width = 9, height = 7,
       units = "in", dpi = 600, device = "pdf")
```

C-index and Integrated Brier Scores: Model performance

```
# combined plot of C-index plot and Brier Scores
## Extracting the data frame
metric3 <- c(rep("C-index" , 4) , rep("Integrated Brier Score" , 4))
model3 <- c(rep(c("Logrank_Mean Model" , "Logrankscore_Mean Model" ,
            "Logrank_Diff Model" , "Logrankscore_Diff Model" ) , 2))
value3 <- c(rsf_perform$result$`C-index`, rsfs_perform$result$`C-index`,
            rsf_perform_d$result$`C-index`, rsfs_perform_d$result$`C-index`,
            rsf_perform$result$`Integrated Brier score`,
            rsfs_perform$result$`Integrated Brier score`,
            rsf_perform_d$result$`Integrated Brier score`,
            rsfs_perform_d$result$`Integrated Brier score`)

Performance_metric3 <- data.frame(metric3, model3, value3)
Performance_metric3$metric3 <- as.factor(Performance_metric3$metric3)
Performance_metric3$model3 <- as.factor(Performance_metric3$model3)
levels(Performance_metric3$model3) <- c("Logrank_Mean Model" ,
                                        "Logrankscore_Mean Model"
                                        ,"Logrank_Diff Model" ,
                                        "Logrankscore_Diff Model")
# the plot
C_Index_IBS_plot3 <- ggplot(Performance_metric3,
                            aes(fill=model3, y=value3, x=metric3)) +
  geom_bar(position="dodge", stat="identity", alpha=0.7) +
  theme_bw() +
  labs(x = "", y = "") +
  theme(axis.text = element_text(size = 15),
        axis.title = element_text(size = 15),
        legend.position = "top", legend.title = element_text(size=14),
        legend.direction = "horizontal",
        legend.text = element_text(size = 10),
        strip.text.x = element_text(size = 15),
        strip.text.y = element_text(size = 15),
        panel.grid.major = element_blank()) +
  guides(fill=guide_legend(title=""))

ggsave("Figure5.pdf", plot = C_Index_IBS_plot3, width = 8, height = 5,
       units = "in", dpi = 600, device = "pdf")
```

Global SHAP values: Average prediction for the data set

```
########## Mean Model ##########
# log-rank
set.seed(32024)
rsf_global_survshap <- model_survshap(explainer = rsf_explainer,
                                      new_observation = testData
                                      [,-which(names(testData) %in%
                                                 c("months", "HIV"))],
                                      y_true =survival::
                                        Surv(testData$months,testData$HIV),
```

```
                                          aggregation_method = "integral",
                                          calculation_method = "kernelshap",)
```

```
# log-rank-score
set.seed(32024)
rsfs_global_survshap <- model_survshap(explainer = rsfscore_explainer,
                                       new_observation = testData
                                       [, -which(names(testData) %in%
                                                    c("months", "HIV"))],
                                       y_true = survival::
                                        Surv(testData$months,testData$HIV),
                                       aggregation_method = "integral",
                                       calculation_method = "kernelshap",)
```

```
########## Difference Model ##########
# log-rank
set.seed(32024)
rsf_global_survshap_d <- model_survshap(explainer = rsf_explainer_d,
                                        new_observation = testData
                                        [, -which(names(testData) %in%
                                                     c("months", "HIV"))],
                                        y_true = survival::
                                        Surv(testData$months,testData$HIV),
                                        aggregation_method = "integral",
                                        calculation_method = "kernelshap",)
```

```
# log-rank-score
set.seed(32024)
rsfs_global_survshap_d <- model_survshap(explainer = rsfscore_explainer_d,
                                         new_observation = testData
                                         [, -which(names(testData) %in%
                                                      c("months", "HIV"))],
                                         y_true = survival::
                                        Surv(testData$months,testData$HIV),
                                        aggregation_method = "integral",
                                        calculation_method = "kernelshap",)
```

Table of average SHAP Values

```
## Combined table
Shap_table <-data.frame(
  Covariates = c('Treatment','Site','Live with partner',
                 'Highest Education','Self Gen Income', 'Salary',
                 'Husband Income','Social Grants','Other Income',
                 'Income Amount','Time in Durban (years)',
                 'Age at enrollment','Marital Status','Age at Debut',
                 'Total Partners','Stable Partners (past year)',
                 'Casual Partners (past year)',
                 'Stable Partners (30 Days)','Casual Partners (30 Days)',
                 'Times Sex (30 Days)','Oldest Partner',
                 'Sex Partner have other','Condom Use',
```

```r
                 'Abnormal Discharge',
                 'Sex Partner test positive',
                 'BASIC_FGF','EOTAXIN','G_CSF','GM_CSF','IFN_G','IL_10',
                 'IL_12P70','IL_13','IL_15','IL_17A','IL_1B','IL_1RA','IL_2',
                 'IL_4','IL_5','IL_6','IL_7','IL_8','IL_9','IP_10','MCP_1',
                 'MIP_1A','MIP_1B','PDGF_BB','RANTES','TNF_A','VEGF','CTACK',
                 'GRO_A','HGF','IFN_A2','IL_12P40','IL_16','IL_18','IL_1A',
                 'IL_2RA','IL_3','LIF','M_CSF','MCP_3','MIF','MIG','SCF',
                 'SCGF_B','SDF_1A','TNF_B','TRAIL','B_NGF'))

#### extracting average SHAP values for log-rank mean model
rsf_surv_shap_values_m <- do.call(cbind, rsf_global_survshap$aggregate)
```

```r
rsf_surv_shap_values_m <- data.frame(unlist(rsf_surv_shap_values_m))
```

```r
rsf_surv_shap_values_m$rsf_shapval_m <- rowMeans(rsf_surv_shap_values_m,
                                                 na.rm = TRUE)
rsf_shapval_mm <- select(rsf_surv_shap_values_m, rsf_shapval_m)
```

```r
#### extracting average SHAP values for log-rank-score mean model
rsfs_surv_shap_values_m <- do.call(cbind, rsfs_global_survshap$aggregate)
```

```r
rsfs_surv_shap_values_m <- data.frame(unlist(rsfs_surv_shap_values_m))
```

```r
rsfs_surv_shap_values_m$rsfs_shapval_m <- rowMeans(rsfs_surv_shap_values_m,
                                                   na.rm = TRUE)
rsfs_shapval_mm <- select(rsfs_surv_shap_values_m, rsfs_shapval_m)
```

```r
#### extracting average SHAP values for log-rank difference model
rsf_surv_shap_values_d <- do.call(cbind, rsf_global_survshap_d$aggregate)
```

```r
rsf_surv_shap_values_d <- data.frame(unlist(rsf_surv_shap_values_d))
```

```r
rsf_surv_shap_values_d$rsf_shapval_d <- rowMeans(rsf_surv_shap_values_d,
                                                 na.rm = TRUE)
rsf_shapval_dd <- select(rsf_surv_shap_values_d, rsf_shapval_d)
```

```r
#### extracting average SHAP values for log-rank-score difference model
rsfs_surv_shap_values_d <- do.call(cbind, rsfs_global_survshap_d$aggregate)
```

```r
rsfs_surv_shap_values_d <- data.frame(unlist(rsfs_surv_shap_values_d))
```

```r
rsfs_surv_shap_values_d$rsfs_shapval_d <- rowMeans(rsfs_surv_shap_values_d, na.rm = TRUE)
rsfs_shapval_dd <- select(rsfs_surv_shap_values_d, rsfs_shapval_d)
```

```r
#### Combining the data frame of the average SHAP values for each model
Shap_table1<- cbind(Shap_table, rsf_shapval_mm, rsfs_shapval_mm, rsf_shapval_dd, rsfs_shapval_dd)

##### extracting to latex code
row.names(Shap_table1) <- NULL
xtable(Shap_table1, digits = 4)
```

Plots for SHAP values

```
########## Mean model ##########
# log-rank
rsf_surv_shap_m <- do.call(rbind, rsf_global_survshap$aggregate)
```

```
rsf_surv_shap_m <- data.frame(unlist(rsf_surv_shap_m))
```

```
rsf_surv_shap_m_long <- rsf_surv_shap_m %>%
  pivot_longer (cols =
                c('Treat','Site','p2v18_REG_PARTNER_LIVE_TOGETHER',
                  'p2v22_HIGHEST_EDUCATION','p3v8_SELF_GEN_INCOME',
                  'p3v9_SALARY','p3v10_HUSBAND','p3v11_SOCIAL_GRANTS',
                  'p3v13_OTHER_INCOME_SOURCE','p3v16_AMOUNT_INCOME',
                  'p5v9_LENGTH_IN_DBNVUL','age1','marital','agedebu',
                  'p17v10_PARTNERS','p17v11_YEAR_STABLE',
                  'p17v12_YEAR_CASUAL','p17v13_30DAYS_STABLE',
                  'p17v14_30DAYS_CASUAL','p17v15_SEX_30DAYS',
                  'p17v26_AGE_OLDEST_SEX_PART',
                  'p17v28_SEX_PART_HAVE_OTHER','p18v15_FREQ_CONDOM_USE',
                  'p19v14_ABNORMAL_DISCHARGE','p18v8_SEX_PART_TEST_POS',
                  'BASIC_FGF','EOTAXIN','G_CSF','GM_CSF','IFN_G','IL_10',
                  'IL_12P70','IL_13','IL_15','IL_17A','IL_1B','IL_1RA',
                  'IL_2','IL_4','IL_5','IL_6','IL_7','IL_8','IL_9',
                  'IP_10','MCP_1','MIP_1A','MIP_1B','PDGF_BB','RANTES',
                  'TNF_A','VEGF','CTACK','GRO_A','HGF','IFN_A2',
                  'IL_12P40','IL_16','IL_18','IL_1A','IL_2RA','IL_3',
                  'LIF','M_CSF','MCP_3','MIF','MIG','SCF','SCGF_B',
                  'SDF_1A','TNF_B','TRAIL','B_NGF'),
              names_to ='var_names', values_to='value') %>%
  arrange(var_names)
rsf_surv_shap_m_long <- rsf_surv_shap_m_long %>%
  dplyr::group_by(var_names) %>% mutate(Med_shap = median(value))

rsf_surv_shap_m_long$fname <-
  str_replace_all(rsf_surv_shap_m_long$var_names, new_names)
rsf_surv_shap_m_long$Direction <- ifelse(rsf_surv_shap_m_long$value > 0,
                                         "Positive", "Negative")
rsf_surv_shap_m_long$var_names <- as.factor(rsf_surv_shap_m_long$var_names)
rsf_surv_shap_m_long$fname <- as.factor(rsf_surv_shap_m_long$fname)
rsf_surv_shap_m_long$Direction <- as.factor(rsf_surv_shap_m_long$Direction)

rsf_surv_shap_plot <- rsf_surv_shap_m_long %>%
  ggplot(mapping = aes(x = value,
                       y = reorder(fname, abs(value), FUN = median),
                       fill = Direction)) +
  geom_boxplot() +
  stat_summary(fun = mean, colour = "darkred", geom = "point") +
  theme_bw() +
  labs(x = "SHAP Value", y = "") +
  theme(
    axis.text = element_text(size = 8),
```

```r
    axis.title = element_text(size = 10),
    legend.position = "top",
    legend.title = element_text(size = 10),
    legend.direction = "horizontal",
    legend.text = element_text(size = 10),
    strip.text.x = element_text(size = 10),
    strip.text.y = element_text(size = 15)
  ) +
  scale_fill_manual(values = c("Positive" = "#90EE90",
                               "Negative" = "#FFA07A")) +
  guides(fill = guide_legend(title = "Direction"))
```

```r
# log-rank-score
rsfs_surv_shap_m <- do.call(rbind, rsfs_global_survshap$aggregate)
```

```r
rsfs_surv_shap_m <- data.frame(unlist(rsfs_surv_shap_m))
```

```r
rsfs_surv_shap_m_long <- rsfs_surv_shap_m %>%
  pivot_longer(cols =
                 c('Treat','Site','p2v18_REG_PARTNER_LIVE_TOGETHER',
                   'p2v22_HIGHEST_EDUCATION','p3v8_SELF_GEN_INCOME',
                   'p3v9_SALARY','p3v10_HUSBAND','p3v11_SOCIAL_GRANTS',
                   'p3v13_OTHER_INCOME_SOURCE','p3v16_AMOUNT_INCOME',
                   'p5v9_LENGTH_IN_DBNVUL','age1','marital','agedebu',
                   'p17v10_PARTNERS','p17v11_YEAR_STABLE',
                   'p17v12_YEAR_CASUAL','p17v13_30DAYS_STABLE',
                   'p17v14_30DAYS_CASUAL','p17v15_SEX_30DAYS',
                   'p17v26_AGE_OLDEST_SEX_PART',
                   'p17v28_SEX_PART_HAVE_OTHER','p18v15_FREQ_CONDOM_USE',
                   'p19v14_ABNORMAL_DISCHARGE','p18v8_SEX_PART_TEST_POS',
                   'BASIC_FGF','EOTAXIN','G_CSF','GM_CSF','IFN_G','IL_10',
                   'IL_12P70','IL_13','IL_15','IL_17A','IL_1B','IL_1RA',
                   'IL_2','IL_4','IL_5','IL_6','IL_7','IL_8','IL_9',
                   'IP_10','MCP_1','MIP_1A','MIP_1B','PDGF_BB','RANTES',
                   'TNF_A','VEGF','CTACK','GRO_A','HGF','IFN_A2',
                   'IL_12P40','IL_16','IL_18','IL_1A','IL_2RA','IL_3',
                   'LIF','M_CSF','MCP_3','MIF','MIG','SCF','SCGF_B',
                   'SDF_1A','TNF_B','TRAIL','B_NGF'),
               names_to ='var_names', values_to='value') %>%
  arrange(var_names)
rsfs_surv_shap_m_long <- rsfs_surv_shap_m_long %>%
  dplyr::group_by(var_names) %>% mutate(Med_shap = median(value))

rsfs_surv_shap_m_long$fname <-
  str_replace_all(rsfs_surv_shap_m_long$var_names,new_names)
rsfs_surv_shap_m_long$Direction <- ifelse(rsfs_surv_shap_m_long$value > 0,
                                          "Positive", "Negative")
rsfs_surv_shap_m_long$var_names <-
  as.factor(rsfs_surv_shap_m_long$var_names)
rsfs_surv_shap_m_long$fname <- as.factor(rsfs_surv_shap_m_long$fname)
rsfs_surv_shap_m_long$Direction <-
  as.factor(rsfs_surv_shap_m_long$Direction)
```

```r
rsfs_surv_shap_plot <- rsfs_surv_shap_m_long %>%
  ggplot(mapping = aes(x = value,
                       y = reorder(fname, abs(value), FUN = median),
                       fill = Direction)) +
  geom_boxplot() +
  stat_summary(fun = mean, colour = "darkred", geom = "point") +
  theme_bw() +
  labs(x = "SHAP Value", y = "") +
  theme(
    axis.text = element_text(size = 8),
    axis.title = element_text(size = 10),
    legend.position = "top",
    legend.title = element_text(size = 10),
    legend.direction = "horizontal",
    legend.text = element_text(size = 10),
    strip.text.x = element_text(size = 10),
    strip.text.y = element_text(size = 15)
  ) +
  scale_fill_manual(values = c("Positive" = "#90EE90",
                               "Negative" = "#FFA07A")) +
  guides(fill = guide_legend(title = "Direction"))
```

```r
Shap_plot1 <- grid.arrange(rsf_surv_shap_plot, rsfs_surv_shap_plot, ncol=2)
ggsave("Figure6.pdf", plot = Shap_plot1,
       width = 8.3, height = 11, units = "in", dpi = 600, device = "pdf")
```

```r
########## Difference model ##########
# log-rank
rsf_surv_shap_d <- do.call(rbind, rsf_global_survshap_d$aggregate)
```

```r
rsf_surv_shap_d <- data.frame(unlist(rsf_surv_shap_d))
```

```r
rsf_surv_shap_d_long <- rsf_surv_shap_d %>%
  pivot_longer(cols=c('Treat','Site','p2v18_REG_PARTNER_LIVE_TOGETHER',
                      'p2v22_HIGHEST_EDUCATION','p3v8_SELF_GEN_INCOME',
                      'p3v9_SALARY','p3v10_HUSBAND','p3v11_SOCIAL_GRANTS',
                      'p3v13_OTHER_INCOME_SOURCE','p3v16_AMOUNT_INCOME',
                      'p5v9_LENGTH_IN_DBNVUL','age1','marital','agedebu',
                      'p17v10_PARTNERS','p17v11_YEAR_STABLE',
                      'p17v12_YEAR_CASUAL','p17v13_30DAYS_STABLE',
                      'p17v14_30DAYS_CASUAL','p17v15_SEX_30DAYS',
                      'p17v26_AGE_OLDEST_SEX_PART',
                      'p17v28_SEX_PART_HAVE_OTHER','p18v15_FREQ_CONDOM_USE',
                      'p19v14_ABNORMAL_DISCHARGE','p18v8_SEX_PART_TEST_POS',
                      'BASIC_FGF','EOTAXIN','G_CSF','GM_CSF','IFN_G','IL_10',
                      'IL_12P70','IL_13','IL_15','IL_17A','IL_1B','IL_1RA',
                      'IL_2','IL_4','IL_5','IL_6','IL_7','IL_8','IL_9',
                      'IP_10','MCP_1','MIP_1A','MIP_1B','PDGF_BB','RANTES',
                      'TNF_A','VEGF','CTACK','GRO_A','HGF','IFN_A2',
                      'IL_12P40','IL_16','IL_18','IL_1A','IL_2RA','IL_3',
                      'LIF','M_CSF','MCP_3','MIF','MIG','SCF','SCGF_B',
                      'SDF_1A','TNF_B','TRAIL','B_NGF'),
```

```r
               names_to='var_names',values_to='value') %>%
  arrange(var_names)

rsf_surv_shap_d_long <- rsf_surv_shap_d_long %>%
  dplyr::group_by(var_names) %>% mutate(Med_shap = median(value))

rsf_surv_shap_d_long$fname <-
  str_replace_all(rsf_surv_shap_d_long$var_names, new_names)

rsf_surv_shap_d_long$Direction <- ifelse(rsf_surv_shap_d_long$value > 0,
                                         "Positive", "Negative")
rsf_surv_shap_d_long$var_names <- as.factor(rsf_surv_shap_d_long$var_names)
rsf_surv_shap_d_long$fname <- as.factor(rsf_surv_shap_d_long$fname)
rsf_surv_shap_d_long$Direction <- as.factor(rsf_surv_shap_d_long$Direction)

rsf_surv_shap_plot_d <- rsf_surv_shap_d_long %>%
  ggplot(mapping = aes(x = value,
                       y = reorder(fname, abs(value), FUN = median),
                       fill = Direction)) +
  geom_boxplot() +
  stat_summary(fun = mean, colour = "darkred", geom = "point") +
  theme_bw() +
  labs(x = "SHAP Value", y = "") +
  theme(
    axis.text = element_text(size = 8),
    axis.title = element_text(size = 10),
    legend.position = "top",
    legend.title = element_text(size = 10),
    legend.direction = "horizontal",
    legend.text = element_text(size = 10), #, angle = 45
    strip.text.x = element_text(size = 10),
    strip.text.y = element_text(size = 15)
  ) +
  scale_fill_manual(values = c("Positive" = "#90EE90",
                               "Negative" = "#FFA07A")) +
  guides(fill = guide_legend(title = "Direction"))
```

```r
# log-rank-score
rsfs_surv_shap_d <- do.call(rbind, rsfs_global_survshap_d$aggregate)
```

```r
rsfs_surv_shap_d <- data.frame(unlist(rsfs_surv_shap_d))
```

```r
rsfs_surv_shap_d_long <- rsfs_surv_shap_d %>%
  pivot_longer(cols = c('Treat','Site','p2v18_REG_PARTNER_LIVE_TOGETHER',
                  'p2v22_HIGHEST_EDUCATION','p3v8_SELF_GEN_INCOME',
                  'p3v9_SALARY','p3v10_HUSBAND','p3v11_SOCIAL_GRANTS',
                  'p3v13_OTHER_INCOME_SOURCE','p3v16_AMOUNT_INCOME',
                  'p5v9_LENGTH_IN_DBNVUL','age1','marital','agedebu',
                  'p17v10_PARTNERS','p17v11_YEAR_STABLE',
                  'p17v12_YEAR_CASUAL','p17v13_30DAYS_STABLE',
                  'p17v14_30DAYS_CASUAL','p17v15_SEX_30DAYS',
                  'p17v26_AGE_OLDEST_SEX_PART',
                  'p17v28_SEX_PART_HAVE_OTHER','p18v15_FREQ_CONDOM_USE',
```

```r
                    'p19v14_ABNORMAL_DISCHARGE','p18v8_SEX_PART_TEST_POS',
                    'BASIC_FGF','EOTAXIN','G_CSF','GM_CSF','IFN_G','IL_10',
                    'IL_12P70','IL_13','IL_15','IL_17A','IL_1B','IL_1RA',
                    'IL_2','IL_4','IL_5','IL_6','IL_7','IL_8','IL_9',
                    'IP_10','MCP_1','MIP_1A','MIP_1B','PDGF_BB','RANTES',
                    'TNF_A','VEGF','CTACK','GRO_A','HGF','IFN_A2',
                    'IL_12P40','IL_16','IL_18','IL_1A','IL_2RA','IL_3',
                    'LIF','M_CSF','MCP_3','MIF','MIG','SCF','SCGF_B',
                    'SDF_1A','TNF_B','TRAIL','B_NGF'),
               names_to='var_names', values_to='value') %>%
  arrange(var_names)

rsfs_surv_shap_d_long <- rsfs_surv_shap_d_long %>%
  dplyr::group_by(var_names) %>% mutate(Med_shap = median(value))

rsfs_surv_shap_d_long$fname <-
  str_replace_all(rsfs_surv_shap_d_long$var_names, new_names)

rsfs_surv_shap_d_long$Direction <- ifelse(rsfs_surv_shap_d_long$value > 0,
                                          "Positive", "Negative")
rsfs_surv_shap_d_long$var_names <-
  as.factor(rsfs_surv_shap_d_long$var_names)
rsfs_surv_shap_d_long$fname <- as.factor(rsfs_surv_shap_d_long$fname)
rsfs_surv_shap_d_long$Direction <-
  as.factor(rsfs_surv_shap_d_long$Direction)

rsfs_surv_shap_plot_d <- rsfs_surv_shap_d_long %>%
  ggplot(mapping = aes(x = value,
                       y = reorder(fname, abs(value), FUN = median),
                       fill = Direction)) +
  geom_boxplot() +
  stat_summary(fun = mean, colour = "darkred", geom = "point") +
  theme_bw() +
  labs(x = "SHAP Value", y = "") +
  theme(
    axis.text = element_text(size = 8),
    axis.title = element_text(size = 10),
    legend.position = "top",
    legend.title = element_text(size = 10),
    legend.direction = "horizontal",
    legend.text = element_text(size = 10),
    strip.text.x = element_text(size = 10),
    strip.text.y = element_text(size = 15)
  ) +
  scale_fill_manual(values = c("Positive" = "#90EE90",
                               "Negative" = "#FFA07A")) +
  guides(fill = guide_legend(title = "Direction"))

Shap_plot2 <- grid.arrange(rsf_surv_shap_plot_d, rsfs_surv_shap_plot_d,
                           ncol=2)
```

```r
# saving high quality figure
ggsave("Figure7.pdf", plot = Shap_plot2,
```

```
       width = 8.3, height = 11, units = "in", dpi = 600, device = "pdf")
```

ROC curves: Model's prediction

```
########## Mean model ##########
# log-rank
set.seed(32024)
rsf_prediction <- predict(rsf_explainer,
                          newdata = testData
                          [, -which(names(testData)%in%
                                      c("months","HIV"))],
                          output_type = "survival")
# log-rank-score
set.seed(32024)
rsfs_prediction <- predict(rsfscore_explainer,
                           newdata = testData
                           [, -which(names(testData) %in%
                                       c("months", "HIV"))],
                           output_type = "survival")
```

```
### year 1
pred_rsf1y <- prediction(1 - rsf_prediction[,12], testData$HIV)
pred_rsfs1y <- prediction(1 - rsfs_prediction[,12], testData$HIV)

perf_rsf1y <- performance(pred_rsf1y, measure = "tpr", x.measure = "fpr")
perf_rsfs1y <- performance(pred_rsfs1y, measure = "tpr", x.measure = "fpr")

RSF_ROC1y <- data.frame(perf_rsf1y@x.values, perf_rsf1y@y.values)
colnames(RSF_ROC1y) <- c("FPR", "TPR")
RSF_ROC1y$Model <- "RSF-logrank"
RSF_ROC1y$Model <- as.factor(RSF_ROC1y$Model)

RSFs_ROC1y <- data.frame(perf_rsfs1y@x.values, perf_rsfs1y@y.values)
colnames(RSFs_ROC1y) <- c("FPR", "TPR")
RSFs_ROC1y$Model <- "RSF-logrankscore"
RSFs_ROC1y$Model <- as.factor(RSFs_ROC1y$Model)

### year 2
pred_rsf2y <- prediction(1 - rsf_prediction[,24], testData$HIV)
pred_rsfs2y <- prediction(1 - rsfs_prediction[,24], testData$HIV)

perf_rsf2y <- performance(pred_rsf2y, measure = "tpr", x.measure = "fpr")
perf_rsfs2y <- performance(pred_rsfs2y, measure = "tpr", x.measure = "fpr")

RSF_ROC2y <- data.frame(perf_rsf2y@x.values, perf_rsf2y@y.values)
colnames(RSF_ROC2y) <- c("FPR", "TPR")
RSF_ROC2y$Model <- "RSF-logrank"
RSF_ROC2y$Model <- as.factor(RSF_ROC2y$Model)

RSFs_ROC2y <- data.frame(perf_rsfs2y@x.values, perf_rsfs2y@y.values)
colnames(RSFs_ROC2y) <- c("FPR", "TPR")
```

```r
RSFs_ROC2y$Model <- "RSF-logrankscore"
RSFs_ROC2y$Model <- as.factor(RSFs_ROC2y$Model)

### combined plot: year 1 & 2
ROC_1Y1 <- rbind(RSFs_ROC1y, RSF_ROC1y)
ROC_1Y1$Time <- "1-Year"
ROC_1Y1$Time <- as.factor(ROC_1Y1$Time)

ROC_2Y1 <- rbind( RSFs_ROC2y, RSF_ROC2y)
ROC_2Y1$Time <- "2-Years"
ROC_2Y1$Time <- as.factor(ROC_2Y1$Time)

ROC_1Y_2Y1 <- rbind(ROC_1Y1, ROC_2Y1)

ROC_1Y_2Y_Plot1 <- ROC_1Y_2Y1 %>%
  ggplot(aes(x = FPR, y = TPR, color = Model)) +
  geom_line(linewidth = 1.5) +
  geom_abline(intercept = 0, slope = 1, color = "grey",
              linetype = "dashed") +
  scale_color_viridis(discrete = TRUE) +
  theme_minimal() +
  labs(x = "False Positive Rate (1 - Specificity)",
       y = "True Positive Rate (Sensitivity)") +
  theme(
    axis.text = element_text(size = 12),
    axis.title = element_text(size = 12),
    legend.position = "top",
    legend.title = element_text(size = 12),
    legend.direction = "horizontal",
    legend.text = element_text(size = 12),
    strip.text.x = element_text(size = 12),
    strip.text.y = element_text(size = 12),
    panel.grid.major = element_blank()
  ) +
  facet_free(. ~ Time, scales = "free") +
  guides(color = guide_legend(title = ""), fill = "none") +
  coord_cartesian(xlim = c(-0.1, 1.1)) +
  scale_x_continuous(breaks = seq(0, 1, 0.25))
```

```r
# saving a high quality figure
ggsave("Figure8.pdf", plot = ROC_1Y_2Y_Plot1,
       width = 5.83, height = 4.13, units = "in", dpi = 600, device = "pdf")
```

```r
########## Difference model ##########
# log-rank
set.seed(32024)
rsf_prediction_d <- predict(
  rsf_explainer_d,
  newdata = testData_d[,
                       -which(names(testData_d) %in% c("months", "HIV"))],
  output_type = "survival")

# log-rank-score
```

```
set.seed(32024)
rsfs_prediction_d <- predict(
  rsfscore_explainer_d,
  newdata = testData_d[,
                      -which(names(testData_d) %in% c("months", "HIV"))],
  output_type = "survival")
```

```
### year 1
pred_rsf1y_d <- prediction(1 - rsf_prediction_d[,12], testData_d$HIV)
pred_rsfs1y_d <- prediction(1 - rsfs_prediction_d[,12], testData_d$HIV)

perf_rsf1y_d <- performance(pred_rsf1y_d, measure = "tpr",
                            x.measure = "fpr")
perf_rsfs1y_d <- performance(pred_rsfs1y_d, measure = "tpr",
                            x.measure = "fpr")

RSF_ROC1y_d <- data.frame(perf_rsf1y_d@x.values, perf_rsf1y_d@y.values)
colnames(RSF_ROC1y_d) <- c("FPR", "TPR")
RSF_ROC1y_d$Model <- "RSF-logrank"
RSF_ROC1y_d$Model <- as.factor(RSF_ROC1y_d$Model)

RSFs_ROC1y_d <- data.frame(perf_rsfs1y_d@x.values, perf_rsfs1y_d@y.values)
colnames(RSFs_ROC1y_d) <- c("FPR", "TPR")
RSFs_ROC1y_d$Model <- "RSF-logrankscore"
RSFs_ROC1y_d$Model <- as.factor(RSFs_ROC1y_d$Model)

### year 2
pred_rsf2y_d <- prediction(1 - rsf_prediction_d[,24], testData_d$HIV)
pred_rsfs2y_d <- prediction(1 - rsfs_prediction_d[,24], testData_d$HIV)

perf_rsf2y_d <- performance(pred_rsf2y_d, measure = "tpr",
                            x.measure = "fpr")
perf_rsfs2y_d <- performance(pred_rsfs2y_d, measure = "tpr",
                             x.measure = "fpr")

RSF_ROC2y_d <- data.frame(perf_rsf2y_d@x.values, perf_rsf2y_d@y.values)
colnames(RSF_ROC2y_d) <- c("FPR", "TPR")
RSF_ROC2y_d$Model <- "RSF-logrank"
RSF_ROC2y_d$Model <- as.factor(RSF_ROC2y_d$Model)

RSFs_ROC2y_d <- data.frame(perf_rsfs2y_d@x.values, perf_rsfs2y_d@y.values)
colnames(RSFs_ROC2y_d) <- c("FPR", "TPR")
RSFs_ROC2y_d$Model <- "RSF-logrankscore"
RSFs_ROC2y_d$Model <- as.factor(RSFs_ROC2y_d$Model)

### combined plot: year 1 & 2
ROC_1Y_d1 <- rbind(RSFs_ROC1y_d, RSF_ROC1y_d)
ROC_1Y_d1$Time <- "1-Year"
ROC_1Y_d1$Time <- as.factor(ROC_1Y_d1$Time)

ROC_2Y_d1 <- rbind(RSFs_ROC2y_d, RSF_ROC2y_d)
ROC_2Y_d1$Time <- "2-Years"
ROC_2Y_d1$Time <- as.factor(ROC_2Y_d1$Time)
```

```
ROC_1Y_2Y_d <- rbind(ROC_1Y_d1, ROC_2Y_d1)

ROC_1Y_2Y_Plot2 <- ROC_1Y_2Y_d %>%
  ggplot(aes(x = FPR, y = TPR, color = Model)) +
  geom_line(linewidth = 1.5) +
  geom_abline(intercept = 0, slope = 1, color = "grey", linetype = "dashed") +
  scale_color_viridis(discrete = TRUE) +
  theme_minimal() +
  labs(x = "False Positive Rate (1 - Specificity)",
       y = "True Positive Rate (Sensitivity)") +
  theme(
    axis.text = element_text(size = 12),
    axis.title = element_text(size = 12),
    legend.position = "top",
    legend.title = element_text(size = 12),
    legend.direction = "horizontal",
    legend.text = element_text(size = 12),
    strip.text.x = element_text(size = 12),
    strip.text.y = element_text(size = 12),
    panel.grid.major = element_blank()
  ) +
  facet_free(. ~ Time, scales = "free") +
  guides(color = guide_legend(title = ""), fill = "none") +
  coord_cartesian(xlim = c(-0.1, 1.1)) +
  scale_x_continuous(breaks = seq(0, 1, 0.25))
```

```
# saving high quality figure
ggsave("Figure9.pdf", plot = ROC_1Y_2Y_Plot2,
       width = 5.83, height = 4.13, units = "in", dpi = 600, device = "pdf")
```

Ablation study

```
################# Mean model ############################
# Log-rank
set.seed(32024)
rsf_logrank <- rfsrc(Surv(months, HIV)~., trainData_top_70,
                     ntree = 500, nodesize = 50, nsplit = 5,
                     splitrule = "logrank", block.size = 1, importance = TRUE)

varimp.rsflr <- gg_vimp(rsf_logrank)
varimp.rsflr <- varimp.rsflr[order(-varimp.rsflr$vimp),]

# Select the first n variable names
top_20_vars <- as.vector(varimp.rsflr$vars[1:20])
top_20_vars1 <- c(top_20_vars, "months", "HIV")
trainData_top_20 <- trainData[, top_20_vars1]

top_30_vars <- as.vector(varimp.rsflr$vars[1:30])
top_30_vars1 <- c(top_30_vars, "months", "HIV")
trainData_top_30 <- trainData[, top_30_vars1]
```

```
top_40_vars <- as.vector(varimp.rsflr$vars[1:40])
top_40_vars1 <- c(top_40_vars, "months", "HIV")
trainData_top_40 <- trainData[, top_40_vars1]

top_50_vars <- as.vector(varimp.rsflr$vars[1:50])
top_50_vars1 <- c(top_50_vars, "months", "HIV")
trainData_top_50 <- trainData[, top_50_vars1]

top_60_vars <- as.vector(varimp.rsflr$vars[1:60])
top_60_vars1 <- c(top_60_vars, "months", "HIV")
trainData_top_60 <- trainData[, top_60_vars1]

top_70_vars <- as.vector(varimp.rsflr$vars[1:70])
top_70_vars1 <- c(top_70_vars, "months", "HIV")
trainData_top_70 <- trainData[, top_70_vars1]

# Log-rank-score
set.seed(32024)
rsf_logrankscore <- rfsrc(Surv(months, HIV)~., trainData_top_70_lrs,
                          ntree = 500, nodesize = 50, nsplit = 5,
                          splitrule = "logrankscore", block.size = 1,
                          importance = TRUE)
varimp.rsflrs <- gg_vimp(rsf_logrankscore)
varimp.rsflrs <- varimp.rsflrs[order(-varimp.rsflrs$vimp),]

# Select the first n variable names
top_20_vars_lrs <- as.vector(varimp.rsflrs$vars[1:20])
top_20_vars1_lrs <- c(top_20_vars_lrs, "months", "HIV")
trainData_top_20_lrs <- trainData[, top_20_vars1_lrs]

top_30_vars_lrs <- as.vector(varimp.rsflrs$vars[1:30])
top_30_vars1_lrs <- c(top_30_vars_lrs, "months", "HIV")
trainData_top_30_lrs <- trainData[, top_30_vars1_lrs]

top_40_vars_lrs <- as.vector(varimp.rsflrs$vars[1:40])
top_40_vars1_lrs <- c(top_40_vars_lrs, "months", "HIV")
trainData_top_40_lrs <- trainData[, top_40_vars1_lrs]

top_50_vars_lrs <- as.vector(varimp.rsflrs$vars[1:50])
top_50_vars1_lrs <- c(top_50_vars_lrs, "months", "HIV")
trainData_top_50_lrs <- trainData[, top_50_vars1_lrs]

top_60_vars_lrs <- as.vector(varimp.rsflrs$vars[1:60])
top_60_vars1_lrs <- c(top_60_vars_lrs, "months", "HIV")
trainData_top_60_lrs <- trainData[, top_60_vars1_lrs]

top_70_vars_lrs <- as.vector(varimp.rsflrs$vars[1:70])
top_70_vars1_lrs <- c(top_70_vars_lrs, "months", "HIV")
trainData_top_70_lrs <- trainData[, top_70_vars1_lrs]

################### Difference model #####################
# Log-rank
set.seed(32024)
```

```r
rsf_logrank_d <- rfsrc(Surv(months, HIV)~., trainData_top_30_d,
                       ntree = 500, nodesize = 50, nsplit = 5,
                       splitrule = "logrank", block.size = 1,
                       importance = TRUE)

varimp.rsflr_d <- gg_vimp(rsf_logrank_d)
varimp.rsflr_d <- varimp.rsflr_d[order(-varimp.rsflr_d$vimp),]

top_20_vars_d <- as.vector(varimp.rsflr_d$vars[1:20])
top_20_vars1_d <- c(top_20_vars_d, "months", "HIV")
trainData_top_20_d <- trainData_d[, top_20_vars1_d]

top_30_vars_d <- as.vector(varimp.rsflr_d$vars[1:30])
top_30_vars1_d <- c(top_30_vars_d, "months", "HIV")
trainData_top_30_d <- trainData_d[, top_30_vars1_d]

top_40_vars_d <- as.vector(varimp.rsflr_d$vars[1:40])
top_40_vars1_d <- c(top_40_vars_d, "months", "HIV")
trainData_top_40_d <- trainData_d[, top_40_vars1_d]

top_50_vars_d <- as.vector(varimp.rsflr_d$vars[1:50])
top_50_vars1_d <- c(top_50_vars_d, "months", "HIV")
trainData_top_50_d <- trainData_d[, top_50_vars1_d]

top_60_vars_d <- as.vector(varimp.rsflr_d$vars[1:60])
top_60_vars1_d <- c(top_60_vars_d, "months", "HIV")
trainData_top_60_d <- trainData_d[, top_60_vars1_d]

top_70_vars_d <- as.vector(varimp.rsflr_d$vars[1:70])
top_70_vars1_d <- c(top_70_vars_d, "months", "HIV")
trainData_top_70_d <- trainData_d[, top_70_vars1_d]

# Log-rank-score
set.seed(32024)
rsf_logrankscore_d <- rfsrc(Surv(months, HIV)~., trainData_top_20_d_lrs,
                            ntree = 500, nodesize = 50,
                            nsplit = 5, splitrule = "logrankscore",
                            block.size = 1, importance = TRUE)

varimp.rsflrs_d <- gg_vimp(rsf_logrankscore_d)
varimp.rsflrs_d <- varimp.rsflrs_d[order(-varimp.rsflrs_d$vimp),]

top_20_vars_d_lrs <- as.vector(varimp.rsflrs_d$vars[1:20])
top_20_vars1_d_lrs <- c(top_20_vars_d_lrs, "months", "HIV")
trainData_top_20_d_lrs <- trainData_d[, top_20_vars1_d_lrs]

top_30_vars_d_lrs <- as.vector(varimp.rsflrs_d$vars[1:30])
top_30_vars1_d_lrs <- c(top_30_vars_d_lrs, "months", "HIV")
trainData_top_30_d_lrs <- trainData_d[, top_30_vars1_d_lrs]

top_40_vars_d_lrs <- as.vector(varimp.rsflrs_d$vars[1:40])
top_40_vars1_d_lrs <- c(top_40_vars_d_lrs, "months", "HIV")
trainData_top_40_d_lrs <- trainData_d[, top_40_vars1_d_lrs]
```

```r
top_50_vars_d_lrs <- as.vector(varimp.rsflrs_d$vars[1:50])
top_50_vars1_d_lrs <- c(top_50_vars_d_lrs, "months", "HIV")
trainData_top_50_d_lrs <- trainData_d[, top_50_vars1_d_lrs]

top_60_vars_d_lrs <- as.vector(varimp.rsflrs_d$vars[1:60])
top_60_vars1_d_lrs <- c(top_60_vars_d_lrs, "months", "HIV")
trainData_top_60_d_lrs <- trainData_d[, top_60_vars1_d_lrs]

top_70_vars_d_lrs <- as.vector(varimp.rsflrs_d$vars[1:70])
top_70_vars1_d_lrs <- c(top_70_vars_d_lrs, "months", "HIV")
trainData_top_70_d_lrs <- trainData_d[, top_70_vars1_d_lrs]

################################################################################
# Explainable Machine learning for Random Survival Forest model
# creating an explainer
########## Mean Model ##########
# log-rank
set.seed(32024)
rsf_explainer <- survex::explain(
  rsf_logrank, data = trainData_top_70[, -which(
    names(trainData_top_70) %in% c("months","HIV"))], y =
    Surv(trainData_top_70$months, trainData_top_70$HIV), label = "RSF-logrank")
rsf_perform <- model_performance(rsf_explainer)
rsf_perform$result$`C-index`

# log-rank-score
set.seed(32024)
rsfscore_explainer <- survex::explain(
  rsf_logrankscore, data = trainData_top_70_lrs[, -which(
    names(trainData_top_70_lrs) %in% c("months","HIV"))], y =
    Surv(trainData_top_70_lrs$months, trainData_top_70_lrs$HIV), label = "RSF-logrankscore")
rsfs_perform <- model_performance(rsfscore_explainer)
rsfs_perform$result$`C-index`

########## Difference Model ##########
# log-rank
set.seed(32024)
rsf_explainer_d <- survex::explain(
  rsf_logrank_d, data = trainData_top_30_d[, -which(
    names(trainData_top_30_d) %in% c("months","HIV"))], y =
    Surv(trainData_top_30_d$months, trainData_top_30_d$HIV), label = "RSF-logrank_d")
rsf_perform_d <- model_performance(rsf_explainer_d)
rsf_perform_d$result$`C-index`

# log-rank-score
set.seed(32024)
rsfscore_explainer_d <- survex::explain(
  rsf_logrankscore_d, data = trainData_top_20_d_lrs[, -which(
    names(trainData_top_20_d_lrs) %in% c("months","HIV"))], y =
    Surv(trainData_top_20_d_lrs$months, trainData_top_20_d_lrs$HIV), label = "RSF-logrankscore_d")
rsfs_perform_d <- model_performance(rsfscore_explainer_d)
rsfs_perform_d$result$`C-index`
```

```
# stop the parallel computation
stopCluster(cl)
```
